# Nature’s contributions to people in mountains: A review

**DOI:** 10.1371/journal.pone.0217847

**Published:** 2019-06-11

**Authors:** Berta Martín-López, Ines Leister, Pedro Lorenzo Cruz, Ignacio Palomo, Adrienne Grêt-Regamey, Paula A. Harrison, Sandra Lavorel, Bruno Locatelli, Sandra Luque, Ariane Walz

**Affiliations:** 1 Leuphana University of Lüneburg, Faculty of Sustainability, Institute for Ethics and Transdisciplinary Sustainability Research, Lüneburg, Germany; 2 Department of Environmental Science, Institute for Wetland and Water Research, Faculty of Science, Radboud University, Nijmegen, The Netherlands; 3 Basque Centre for Climate Change (BC3), Bilbao, Spain; 4 Social-Ecological Systems Laboratory, Universidad Autónoma de Madrid, Madrid, Spain; 5 Planning of Landscape and Urban Systems PLUS, Swiss Federal Institute of Technology (ETH), Zurich, Switzerland; 6 Centre for Ecology & Hydrology, Lancaster Environment Centre, Library Avenue, Bailrigg, United Kingdom; 7 Laboratoire d’Ecologie Alpine, Centre National de la Recherche Scientifique (CNRS) and Université Grenoble-Alpes, Grenoble, France; 8 Agricultural Research for Development (CIRAD), University of Montpellier, Montpellier, France; 9 Centre for International Forestry Research (CIFOR), Lima, Peru; 10 National Research Institute of Science and Technology for Environment and Agriculture (IRSTEA), UMR TETIS, Montpellier, France; 11 University of Potsdam, Institute of Earth and Environmental Science, Potsdam, Germany; University of New England, AUSTRALIA

## Abstract

Mountains play a key role in the provision of nature’s contributions to people (NCP) worldwide that support societies’ quality of life. Simultaneously, mountains are threatened by multiple drivers of change. Due to the complex interlinkages between biodiversity, quality of life and drivers of change, research on NCP in mountains requires interdisciplinary approaches. In this study, we used the conceptual framework of the Intergovernmental Science-Policy Platform on Biodiversity and Ecosystem Services (IPBES) and the notion of NCP to determine to what extent previous research on ecosystem services in mountains has explored the different components of the IPBES conceptual framework. We conducted a systematic review of articles on ecosystem services in mountains published up to 2016 using the Web of Science and Scopus databases. Descriptive statistical and network analyses were conducted to explore the level of research on the components of the IPBES framework and their interactions. Our results show that research has gradually become more interdisciplinary by studying higher number of NCP, dimensions of quality of life, and indirect drivers of change. Yet, research focusing on biodiversity, regulating NCP and direct drivers has decreased over time. Furthermore, despite the fact that research on NCP in mountains becoming more policy-oriented over time, mainly in relation to payments for ecosystem services, institutional responses remained underexplored in the reviewed studies. Finally, we discuss the relevant knowledge gaps that should be addressed in future research in order to contribute to IPBES.

## 1. Introduction

Mountains provide ecosystem services that contribute to the wellbeing of people living in them or their foothills (around 20% of the world’s population) and many more in the adjacent lowlands [1]. Mountains occupy 24% of the global land surface [2] and host the world’s principal biomes supplying a diverse array of ecosystem services [3,4]. They play a key role in the water cycle as their grasslands, wetlands and forests contribute to water flow regulation and water quality improvement through water storage and infiltration, as well as the capture of atmospheric vapour (e.g. by mountainous tropical cloud forests) [5]. As mountains are the source of the world’s major rivers, more than half of world’s population depends on freshwater regulated and purified by upstream mountain ecosystems [6]. Mountain ecosystems also regulate natural hazards, such as avalanches, landslides and rock falls, which can be ameliorated by their forests [7,8]. Mountain ecosystems produce material benefits that make substantial contributions to lowland and highland economies, such as crops often from subsistence agriculture, animal products from grazing activities, timber, fuelwood, and non-timber products, including game and medicinal plants. In addition, mountains are important providers of non-material benefits because of their scenic beauty and biodiversity [4,9–13]. Mountains are major destinations for touristic or recreation activities and host sites and species of important heritage and cultural values [14,15]. Such cultural values are associated with symbolic and spiritual feelings, sense of identity and place, wonder and respect, and the mental wellbeing of local population and visitors [16].

However, mountain ecosystem services are being affected by various drivers of change, such as rural abandonment, climate and land-use change [4,17–20]. In particular, mountain landscapes offer opportunities to observe early impacts of climate change on ecosystem services [18]. For instance, rapid glacier retreat and loss of snow cover in the Himalayan Mountains has important consequences on water availability for ecosystems and people within and far beyond mountain boundaries [21]. Climate change also alters the distribution of plant species and ecosystem functioning, with important implications for the long-term provision of ecosystem services essential for the wellbeing of many people [22]. Increasing natural hazards, such as rock fall, debris flows and floods, threaten residents and visitors of mountain areas and destroy critical infrastructure [17]. These climate-induced changes are further superimposed and accelerated by demographic and socio-economic changes, changing the supply and demand of ecosystem services, and ultimately impacting economic activities in mountain areas [22].

Due to the multiple and complex interlinkages between ecosystem services, biodiversity, human wellbeing and drivers of change [23,24], greater collaboration across the humanities, social sciences and natural sciences is required [25]. A truly integrated and interdisciplinary research approach is essential for studying the interactions between socio-economic and ecological systems in mountains, including ecosystem services and nature’s contributions to people (NCP) [25].

**Fig 1:**
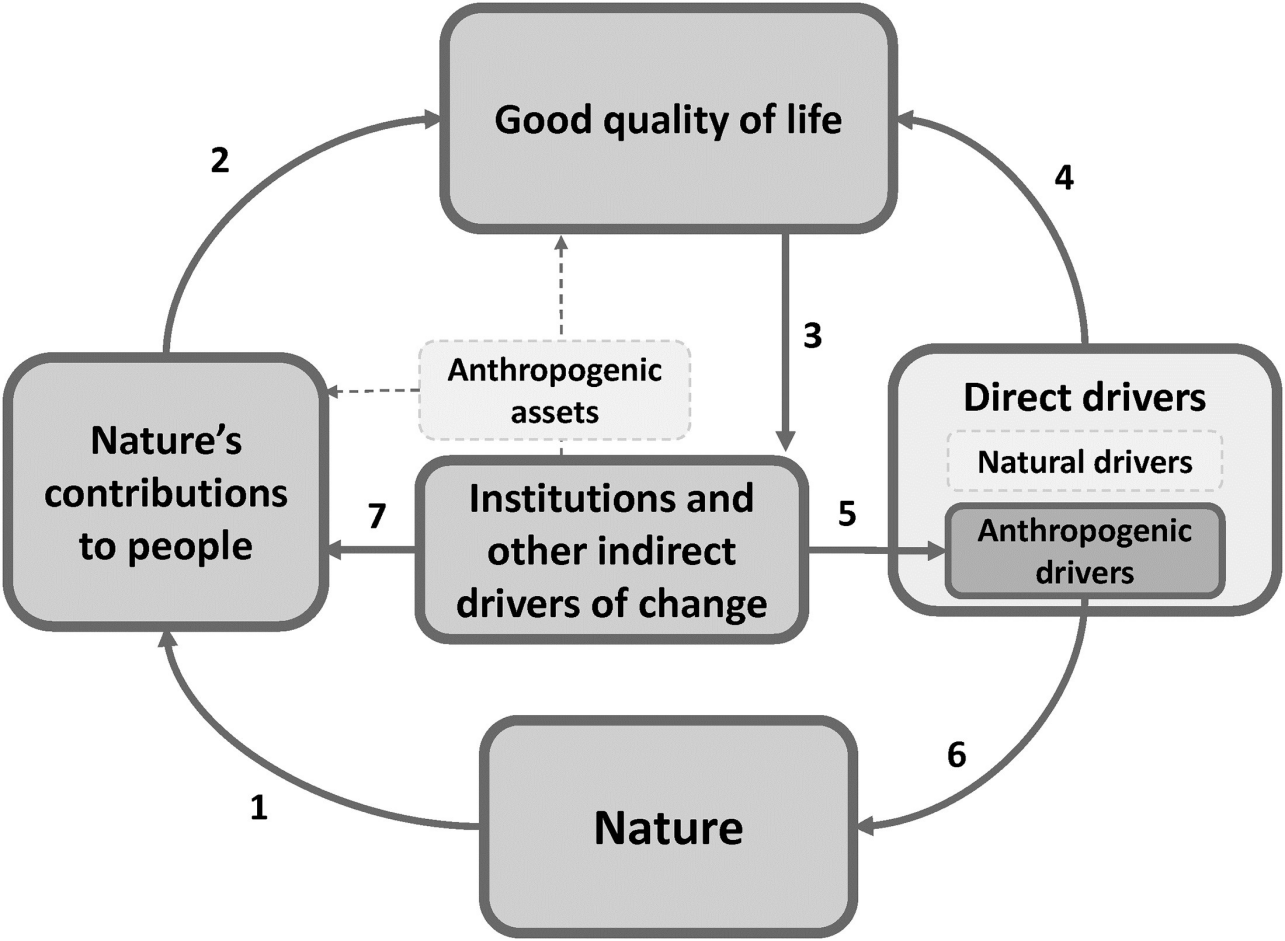
The IPBES conceptual framework. Boxes denote the basic components of nature and society that are the focus of IPBES and dark grey boxes with non-dotted lines indicate those components that are the focus of this research: nature, nature’s contributions to people (after Díaz et al. (2018) [34]), good quality of life, direct drivers of change, and indirect drivers of change, with particular emphasis on governance. The interactions between these components analyzed in this study are presented with non-dotted arrows. The numbered arrows between the elements represent influences and interactions between IPBES components that are researched in this study. Only those inclusive categories referring to each of the components are shown in the diagram, but see Díaz et al. (2015) [24] for the categories referring to western science and indigenous and local knowledge.

Among the existing frameworks to analyze complex interactions between socio-economic and ecological systems, the recent conceptual framework of the Intergovernmental Science-Policy Platform on Biodiversity and Ecosystem Services (IPBES) offers a clear synthesis of the interlinkages between people and nature [24]. This framework has been successfully applied in the IPBES Regional Assessments on Biodiversity and Ecosystem Services [26–29] and the IPBES Thematic Assessments on pollinators, pollination and food production [30] and on land degradation and restoration [31], as well as in research derived from these assessments (e.g. [32]) and socio-cultural valuation research (e.g. [33]). The conceptual framework of IPBES considers six main components that represent the social and ecological systems as well as their interactions (see Fig 1): nature, NCP, good quality of life, anthropogenic assets, direct drivers of change, and indirect drivers of change, with particular emphasis on governance [24].

Nature refers to the living organisms and their interactions among themselves and with their environment [24] and relates to species, ecological communities, functional diversity and biodiversity as a general concept. Nature contributes to the good quality of people’s life by providing beneficial NCP, including material, regulating and non-material contributions [24,34]. Good quality of life refers to the accomplishment of a fulfilled life by humans [24] and encompasses access to basic materials, health, good social relationships, security and freedom of choice [23,24]. Direct drivers of change are endogenous pressures of the system that alter the state of nature and people’s quality of life [35]. Anthropogenic direct drivers of change include land-use changes, climate change, pollution, species introductions and overexploitation of resources [23,24]. Indirect drivers of change refer to those underlying causes of changes that are exogenous to the social-ecological system, such as changes in economy, demography, culture or lifestyles [23,24,36]. The conceptual framework of IPBES emphasizes the key role of institutions as they determine the access to and control over NCP (Fig 1). Moreover, people’s quality of life can trigger institutional responses in the form of management actions aiming to either minimize the negative impacts of drivers of change or maximise the provision of beneficial NCP (Fig 1) [19,36,37].

As mountains are essential providers of NCP [3,4] and face multiple drivers of change [4], examining previous research on NCP is important for identifying knowledge gaps and ways to design future research agendas. In this paper, we analyze to what extent former research on ecosystem services conducted in mountains has explored the different components of the IPBES conceptual framework and social-ecological interactions with interdisciplinary lenses. We finally discuss the most important research gaps and ways to move forward the research agenda of NCP in mountains in order to support IPBES.

## 2. Methods

We conducted a systematic review of peer-reviewed scientific articles written in English on ecosystem services in mountainous regions worldwide using the Web of Science and Scopus databases. We followed the guidelines of the Preferred Reporting Items for Systematic Reviews and Meta-Analyses (PRISMA) for conducting systematic reviews. PRISMA was published to set standards in the reporting of systematic reviews in 2009 [38] and has proved essential for increasing both reporting and methodological quality [39]. The PRISMA checklist of rules for this study is given in S1 Table.

To avoid double counting, we searched for primary research articles and excluded reviews and conference proceedings. The search string used for the review comprised three main elements: (1) mountainous systems (e.g. mountain, highland, *paramo* or summit) and regions (e.g. Andes, Alps, Himalaya, Ghats or Kilimanjaro), (2) assessment and valuation (e.g. evaluation, assessment, valuation, mapping, quantification or estimation) and (3) ecosystem services (e.g. ecosystem service, ecosystem good, environmental service or ecosystem function). We included in the search string the term ‘ecosystem function’ to find publications that researched the benefits derived from ecosystem functions, thus qualifying as ecosystem services, without mentioning the term ‘ecosystem service’ or ‘environmental service’. Although we used the IPBES framework, it is important to point out that the terms of ‘nature’s contributions to people’ and ‘nature’s benefits to people’ (see [24]) have not been used yet in mountains research and therefore their use in the search string does not provide any results. For the full search string, see S1 Text.

The search was applied to Abstract, Title and Keywords of published papers between 1997 and 2016. The search returned 580 papers (S1 Fig). Based on the screening of titles and abstracts, we disregarded 60 papers that were conceptual, theoretical or reviews or were not conducted in mountains (S1 Fig). In a second step, we assessed the eligibility of the papers by reading the full text. In this step, besides the same criteria used in the first screening, we also excluded those papers whose full text was not available in English, and those that mentioned the term ‘ecosystem service’ without assessing, valuing or mapping any ecosystem service. A final set of 213 papers were selected for in-depth analyses (S1 Fig). S2 Text provides the final list of 213 papers included in this research. As we are aware that ecosystem service research is a fast evolving field with increasing numbers of publications each year, we also reviewed 10% of the 310 papers that met the screening criteria of a total of 413 papers that were returned after applying the search methodology to papers published between 2017 and 2018. Analyses of these additional papers was undertaken to confirm that the patterns of ecosystem service research found in this study are consistent with current literature from 2017–2018. S3 Text provides a synthesis of the results of the review of 10% of the papers for 2017 and 2018.

The information extracted from each paper was organized in a database according to (1) characteristics of the publication (i.e. year of publication and journal); (2) study location; (3) methodological approach (i.e. biophysical, socio-cultural, economic and plural approaches, which included different methods from natural and social sciences [40]); (4) component of the IPBES framework analyzed; (5) the element of nature (i.e. species, community, functional diversity and biodiversity in its broad sense); (6) NCP categories; (7) dimensions of good quality of life; (8) direct drivers of change; (9) indirect drivers of change; and (10) institutional responses, particularly if they referred to protected areas, legislation or market-based schemes, such as payments for ecosystem services. Although institutional responses are also indirect drivers of change, in this paper we distinguished between the two: while indirect drivers of change refer to those policy changes that have jeopardized the capacity of mountains to continue providing NCP, institutional responses refer to those management actions that deliberately seek to preserve NCP. This distinction also aligns with the IPBES conceptual framework since it emphasizes the key role of institutional responses to manage and preserve nature and NCP [24].

To classify NCP, we used the 18 categories of the IPBES generalizing perspective [34]. These categories were grouped into regulating, material and non-material NCP [34]. It is important to note that, as we reviewed the literature on ecosystem services, we only considered those beneficial or positive NCP. Despite the initial debate about the added value of NCP in current research [41–43], we consider that it can provide useful insights for fostering future research in mountains for two reasons. First, as the paradigm of NCP considers local ecological knowledge as a relevant source of information [34], its application can help to (1) reveal benefits that have been overlooked in scans of previous research on the topic and (2) foster socio-cultural approaches that have been less developed in ecosystem service research [44–47]. For example, drawing on the local knowledge of shepherds in different mountain ranges of Spain, Morales-Reyes et al. [48] found that carcass removal provided by large carnivores and vultures was an essential beneficial contribution that reduces the economic costs of farms. To date, the beneficial NCP of scavenging has not been considered in standard classifications of ecosystem services, including those from the Millennium Ecosystem Assessment [23], the Economics of Ecosystems and Biodiversity [49] and the Common International Classification of Ecosystem Services [50]. Therefore, while encompassing the ecosystem services framework, the paradigm of NCP enables new beneficial and detrimental contributions that have been historically overlooked in former classifications of ecosystem services to be uncovered. In addition, the paradigm of NCP acknowledges that one particular contribution can simultaneously belong to the different categories of material, non-material and regulating. This has special relevance for mountains as particular contributions such as gathering of wild food (e.g. mushrooms, asparagus or berries), hunting and fishing can be considered both material and non-material NCP because they provide nutritional benefits and recreational enjoyment [51].

Dimensions of good quality of life included fulfilling the needs of basic materials (i.e. ability to access resources to gain a livelihood and earn income), health, security, good social relationships, and freedom of choice and action [23]. Direct drivers of change were classified as land-use change, climate change, overexploitation of natural resources, invasive alien species and pollution [23,24] and indirect drivers of change were classified as policy changes (i.e. changes in conservation and agricultural policies), changes in cultural values, demographic changes (i.e. rural abandonment and population growth), and market forces [23].

We conducted network analysis to explore to what extent interactions between the different abovementioned components of the IPBES framework were analyzed in the scientific literature of ecosystem services provided by mountains. In the networks, nodes represent the biodiversity levels, categories of NCP, dimensions of wellbeing, direct and indirect drivers of change and institutional responses. Then, when two specific components were analyzed together in the same publication, we paired them using undirected links. The interlinkages between the nodes (i.e. edges) represent the number of co-occurrences between the different components of the IPBES framework. Thus, the weight of edges was calculated according to the number of papers in which a pair of nodes appears together [52].

**Fig 2:**
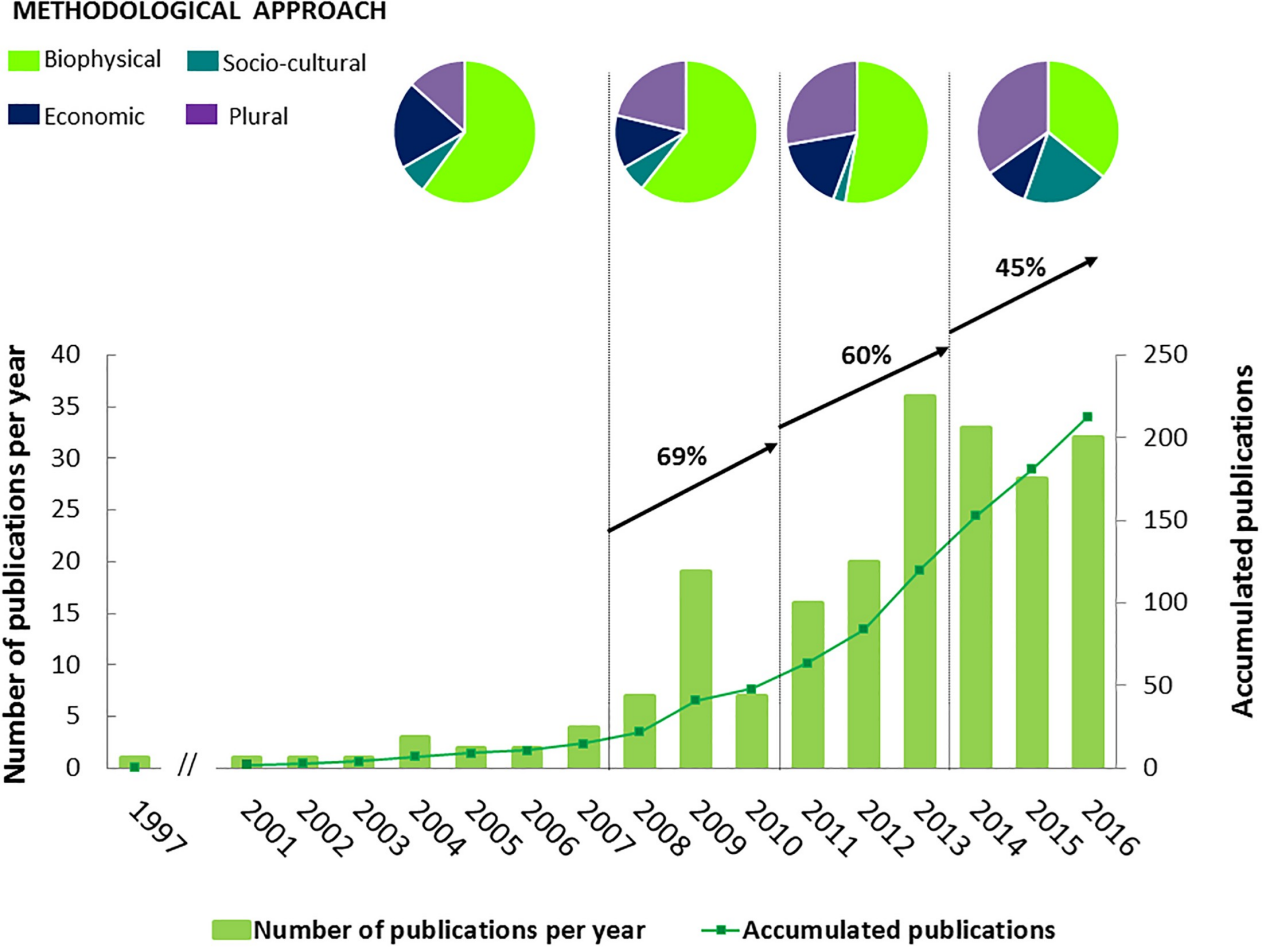
Temporal distribution of papers published on ecosystem services in mountains and main methodological approach applied in each period (1997–2007, 2008–2010, 2011–2013, 2014–2016).

This process resulted in four undirected weighted networks, which were built according to four periods of time: 1997–2007, 2008–2010, 2011–2013, and 2014–2016. These periods were selected after analyzing historical trends in ecosystem services publications, in particular the length of each period (i.e. 3 years, except the first period which is an open interval: 2007 and before) and a similar growth rate of publications in each period (which ranges between 45% and 69%) (Fig 2). To determine to what extent the nodes representing each IPBES component were relevant in shaping ecosystem service research on mountains in each time period, we calculated two measures of centrality: weighted degree and betweenness. The weighted degree of a given node measures the sum of the weights (i.e., numbers of publications in our analysis) of the edges connecting this node to the others [53]. Betweenness measures how many times a node relates to other nodes which would be otherwise disconnected [54,55]. Therefore betweenness for a particular component of the IPBES framework (node) is measured as the number of shortest paths that pass through this node divided by all existing shortest paths in the network [52]. This centrality metric is thus able to identify which specific components of the IPBES framework act as linkages and connect otherwise disparate components. Along with the centrality metrics, we also calculated the density of the network, which is the proportion of node pairs out of the total number of possible pairs in the network [52]. Thus, density indicates to what extent the research on ecosystem services provided by mountains included the interlinkages between the different components of the conceptual framework of IPBES. We created the networks and calculated the centrality metrics using the NodeXL and Gephi software.

## 3. Results

### 3.1. Temporal trends and geographical distribution

The volume of scientific papers on ecosystem services in mountains (reviewed in this research) has rapidly increased since 2008, consistent with the general development of ecosystem service research. Four periods can be distinguished within the overall trend (Fig 2): (1) from 1997 to 2007 (a period comprising 10 years of research and a total number of 15 articles), when there was an average of 2 publications per year, (2) from 2008 to 2010 (a period comprising 3 years and a total number of 33 articles), when research reached a peak with 19 papers published in 2009, (3) from 2011 to 2013 (a period comprising 3 years and a total number of 72 articles), when the number of publications increased from 16 to 36 papers per year, and (4) from 2014 to 2016 (a period comprising 3 years and a total number of 93 articles), the number of published papers per year stabilized around 30 (2014: n = 33; 2015: n = 28 and 2016: n = 32 papers) (Fig 2). We also found that these four periods are characterized by different methodological approaches for assessing ecosystem services (Fig 2). Whilst in the first and second periods more than 60% of the papers (*n* = 9 and *n* = 20, respectively) applied biophysical methods, this decreased to 36% in 2014–2016 (*n* = 38). By contrast, socio-cultural methodological approaches increased from 7% and 6% of publications in the first and second periods (*n* = 1 and *n* = 2), respectively, to 20% in the fourth period (*n* = 18). Economic valuation methods were mainly applied before 2007 (20% of publications; *n* = 3) and in 2010–2013 (16% of publications; *n* = 12). Plural and integrated assessments, in which different methodological approaches were applied, gradually increased from 13% of publications (*n* = 2) before 2007 to 35% of publications (*n* = 32) in 2014–2016. Overall, biophysical assessment methods were the most applied (47% of publications; *n* = 100), followed by plural and integrated assessments (29%; *n* = 61), economic valuation (13%; *n* = 28) and socio-cultural approaches (11%; *n* = 23).

**Fig 3:**
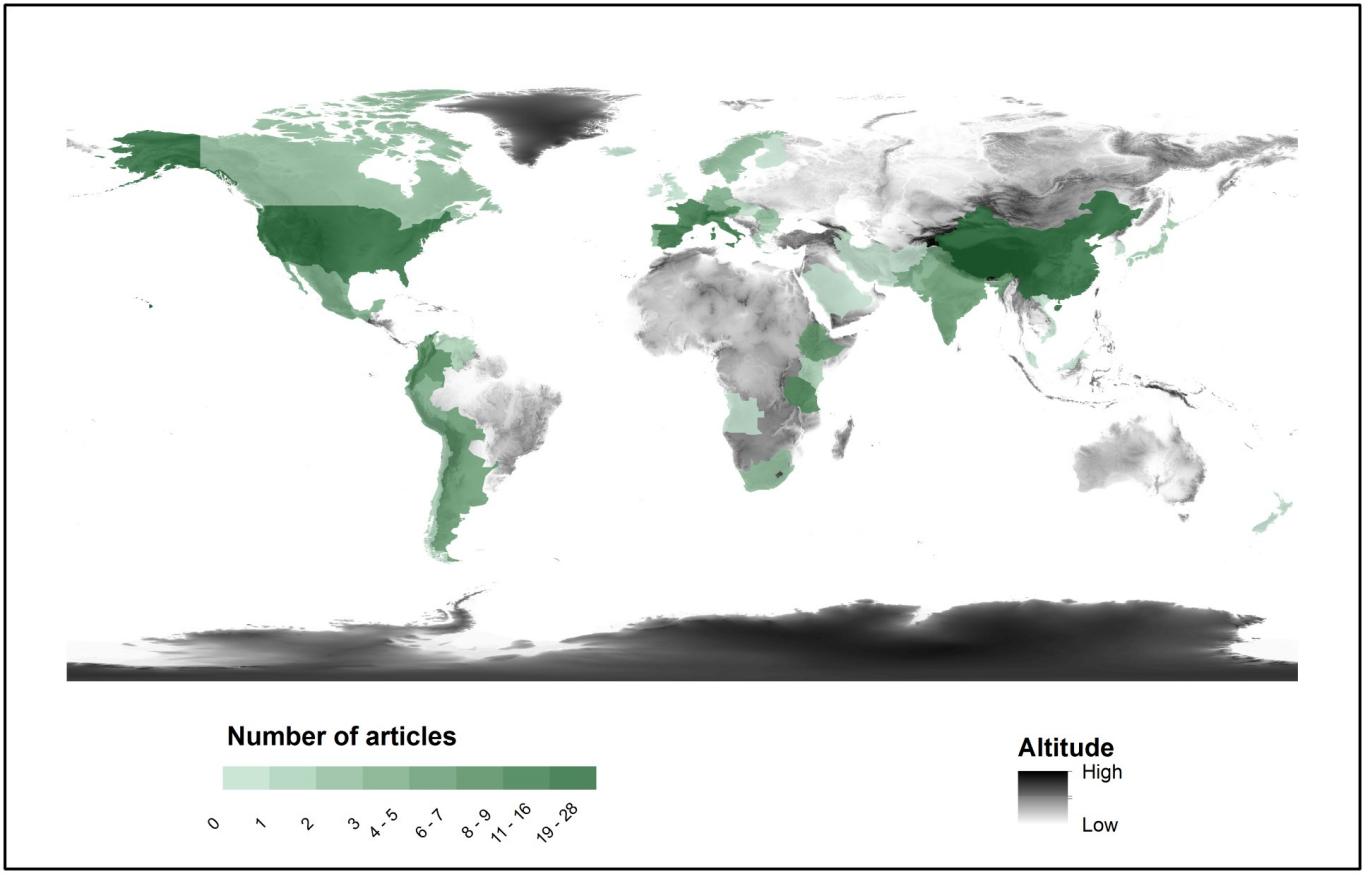
Geographic distribution of papers published on ecosystem services provided by mountains.

The studies addressing ecosystem services in mountains are unevenly distributed geographically (Fig 3). The largest part of the studies focused on European mountains (34.9% of publications; *n* = 74), mostly in Switzerland (26.0% of European publications; *n* = 19), Italy (21.9%; *n* = 16), France (20.5%; *n* = 15) and Spain (15.1%; *n* = 11). However, the country with the highest number of publications is China (13.4% of publications; *n* = 28), followed by the United States (10.0%; *n* = 21). By contrast, the Andes in Latin America presented few studies (8.9% of publications; *n* = 19) relative to their extension, as well as the South draining parts of the Himalayas and Central Asian mountain ranges (Fig 3). Moreover, we did not find studies addressing ecosystem services in other mountain ranges, such as the Ural Mountains and the Armenian and the Anatolian Plateaus (Fig 3).

### 3.2. Coverage of different components of social-ecological systems

**Fig 4:**
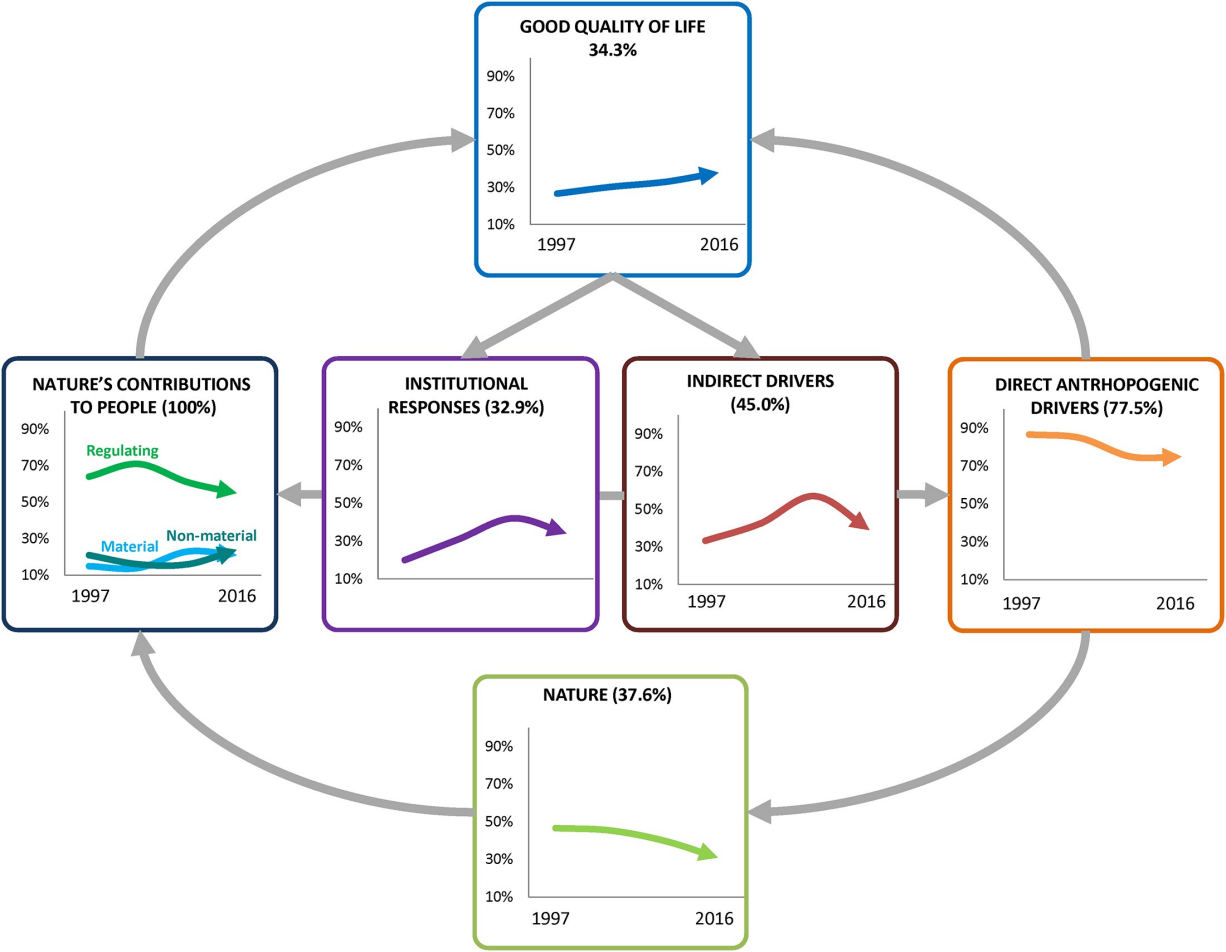
Temporal trends in the focus on published research on mountain ecosystem services according to the components of the IPBES conceptual framework.

**Fig 5:**
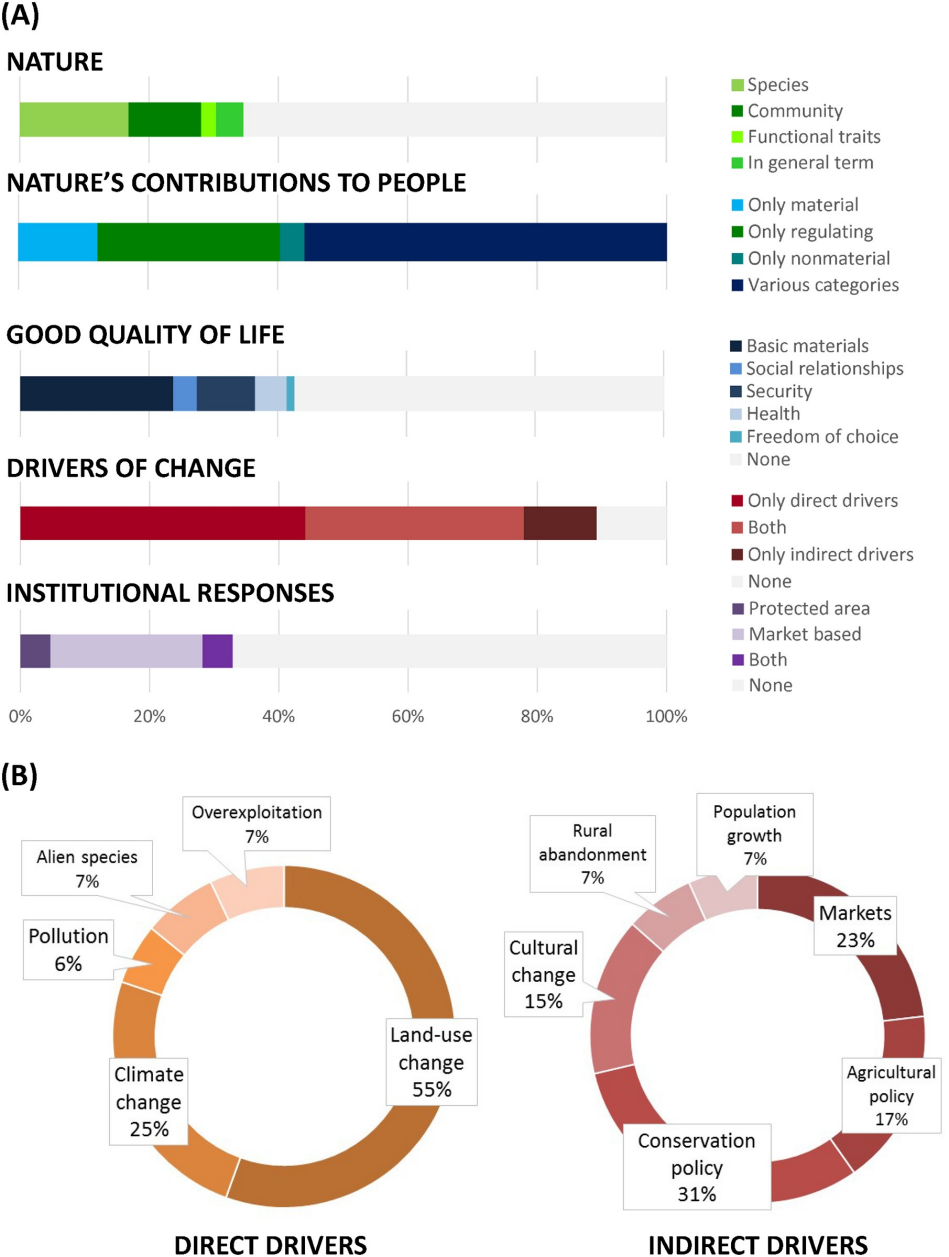
Percentage of publications on ecosystem services provided by mountains that focus on the different components of the IPBES conceptual framework. (A) Percentage of publications that analyze different aspects of nature, categories of NCP, components of human wellbeing, and types of drivers of change. (B) Percentage of the studies analyzing different direct drivers (left) and indirect drivers (right).

Research on ecosystem services in mountains covered the component of nature in 37.6% of publications (*n* = 80), with the coverage decreasing across the four time periods (Fig 4). Amongst the organizational levels of biodiversity, 16.9% of publications (*n* = 36) evaluated the level of species or populations, followed by the community level (11.3% of publications; *n* = 24) (Fig 5A). Only 2.3% of publications (*n* = 5) approached biodiversity from the functional diversity perspective and 4.2% (*n* = 9) referred to biodiversity in its broad sense (Fig 5A).

Fifty-six percent of publications (*n* = 119) assessed more than one category of NCP (Fig 5A). From the remaining 44.2% of publications, 28.2% (*n* = 60) assessed only regulating NCP, 12.2% (*n* = 26) assessed only material NCP and 3.8% (*n* = 8) focused only on non-material NCP. Despite regulating NCP being the category that received the most scientific attention overall, their proportion decreased across the four time periods (Fig 4). By contrast, research on material and non-material NCP slightly increased (Fig 4). The NCP that received the highest scientific attention were physical and psychological experiences (50.7% of publications; *n* = 108), which encompassed recreational experiences (30.5% of publications; n = 65) and aesthetic enjoyment (20.2%; n = 43) (S2 Fig). The category of supporting identities (29.1% of publications; *n* = 62) (S2 Fig) was also a frequently assessed non-material NCP. This category encompassed supporting cultural identity and sense of belonging (16.4% of publications; *n* = 35), the satisfaction derived from knowing that a particular landscape and species exists (7.0%; *n* = 15) and supporting sacred and spiritual rituals (5.6%; *n* = 12). The regulating NCP that received the highest scientific attention was regulation of climate (45.5%; *n* = 97), followed by soil formation (41.8% of publications; *n* = 89), regulation of freshwater quality (39.4%; *n* = 84) and quantity (32.9%; *n* = 70) and habitat maintenance (31.9%; *n* = 68) (S2 Fig). Provision of food and feed (46.0%; *n* = 98) was the material NCP that received the highest scientific attention, followed by the provision wood and fibres (32.8% and 30.5% of publications; *n* = 70 and *n* = 65, respectively) (S2 Fig).

Quality of life was only assessed in 34.3% of publications (*n* = 73) on ecosystem services in mountains, although it presented an increasing trend from 26.7% of the articles (*n* = 57) in the period 1997–2007 to 38.0% in 2014–2016 (*n* = 81) (Fig 4). Amongst the dimensions of good quality of life, the category of basic materials received the highest scientific attention (27.2% of publications; *n* = 58), followed by security (10.3%; *n* = 22). Nature’s contributions to health and good social relationships were rarely assessed (5.6% and 4.2%; *n* = 19 and *n* = 9, respectively) (Fig 5A).

Impacts of drivers of change on nature and NCP were assessed very frequently, with 89.2% of publications (*n* = 190): 33.8% of publications (*n* = 72) assessed both direct and indirect drivers of change, 44.1% of publications (*n* = 94) assessed only direct drivers and 11.3% of publications (*n* = 24) assessed only indirect drivers (Fig 5A). The trend of assessing both drivers of change was relatively stable across the four periods of time, from 33.3% in the 1997–2007 period (*n* = 5) to 31.2% in the 2014–2016 period (*n* = 29). Although direct drivers of change were assessed in 77.5% of the publications (*n* = 165), their prevalence declined across the four time periods (Fig 4) because publications assessing only direct drivers of change decreased from 53.3% in the 1997–2007 period (*n* = 8) to 44.1% in the 2014–2016 period (*n* = 41). By contrast, the analysis of indirect drivers of change increased from 33.3% of publications in the 1997–2007 period (*n* = 5) to 57.0% of publications in 2010–2013 (*n* = 41) (Fig 4). The number of publications assessing only indirect drivers of change increased from 0% in the 1997–2007 period to 19.4% of publications in 2010–2013 (*n* = 14) and 7.5% of publications in 2014–2016 (*n* = 7). Most of the research about the impacts of direct drivers of change on NCP has focused on land-use change (55.5% of publications that analyzed direct drivers; *n* = 126) and climate change (24.7%; *n* = 56) (Fig 5B). Regarding indirect drivers, 31.1% of the publications (*n* = 51) explored the impact of current conservation policies on nature and NCP, followed by the impact of markets (23.2% of publications on indirect drivers; *n* = 38), agricultural policies (17.1%; *n* = 28) and cultural change (15.2%; *n* = 25). Demographic changes, either emigration from rural areas or human population growth, were rarely covered in the literature (6.7% of publications each; *n* = 11) (Fig 5B).

Finally, institutional responses to preserve nature and NCP in mountains were only addressed by 32.9% of publications (*n* = 70) (Fig 4). The consideration of conservation measures, both protected areas and market-based instruments (e.g. payments for ecosystem services, habitat banking) increased over the four time periods from 20.0% in 1997–2007 (*n* = 3) to 34.3% in 2014–2016 (*n* = 32) (Fig 4). Particularly, the consideration of market-based schemes increased from 6.6% of publications in the 1997–2007 period (*n* = 1) to 30.5% and 29.0% of publications in 2010–2013 and 2014–2016 (*n* = 22 and *n* = 27), respectively.

### 3.3. Interrelations between social-ecological system components

**Fig 6:**
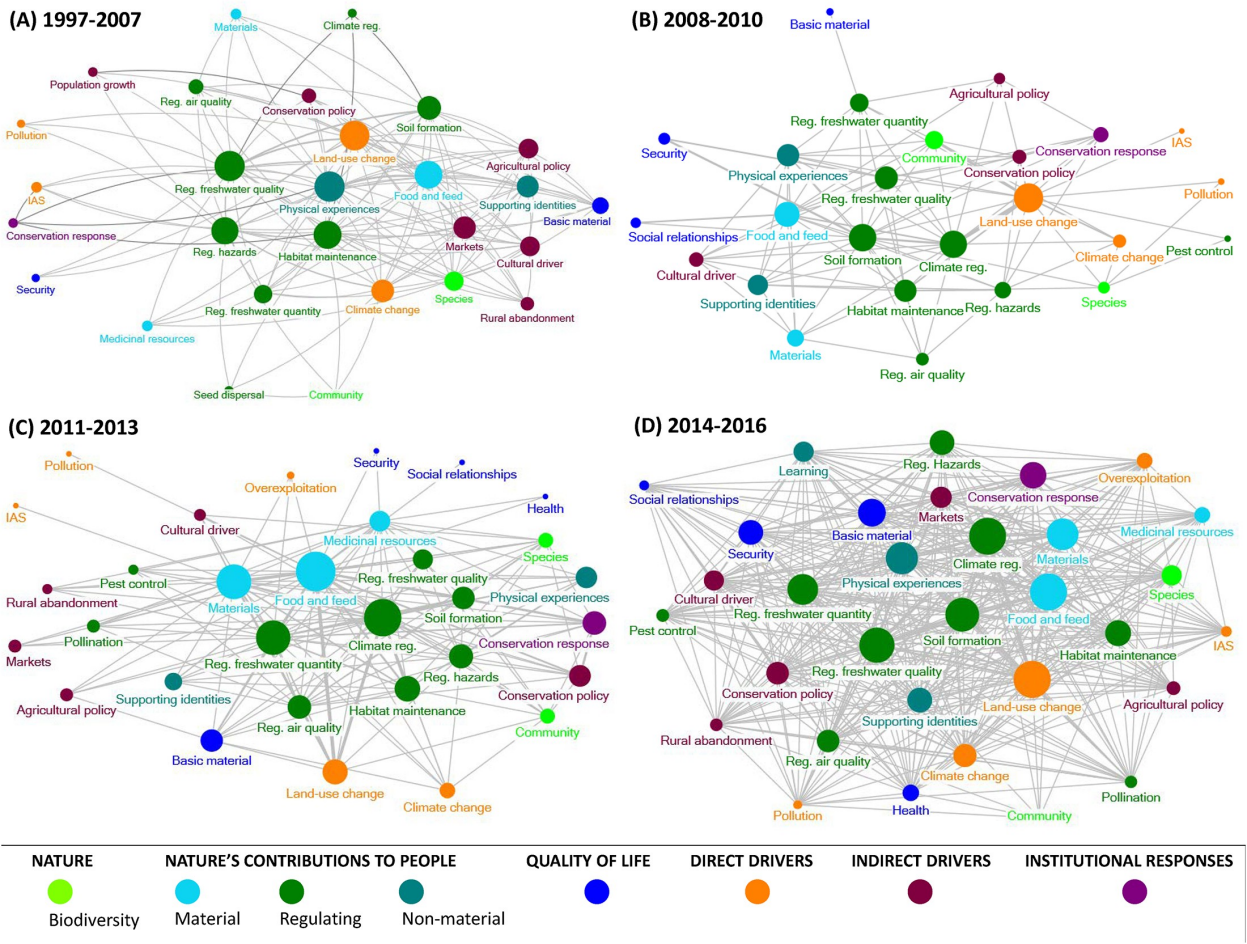
Network of different components of the IPBES conceptual framework that have been addressed in papers published about ecosystem services provided by mountains in the four periods identified: 1997–2007, 2008–2010, 2011–2013, 2014–2016. Symbol size indicates weighted degree of nodes (maximum weighted degrees: 78 (period 1997–2007), 250 (2008–2010), 250 (2011–2013), 872 (2014–2016). Symbol color indicates the different components of the IPBES framework. Width of edges (gray lines) represent edge weights, i.e. the number of papers (maximum weight 76, only edges with weights higher than 2 are shown).

Research on ecosystem services in mountains has increasingly considered the complex interactions between indirect and direct drivers, nature, NCP, human wellbeing and institutional responses over the four time periods (Fig 6). Thus, the density of the networks has slightly increased over the first three periods, i.e. 1997–2007 (0.171), 2008–2010 (0.282), 2011–2013 (0.296), and is markedly higher in the last period, i.e. 2014–2016 (0.800). The number of components (nodes) in each of the IPBES components has slighted increased over time (from 36 before 2007 to 41 after 2014), particularly through the inclusion of the wellbeing dimensions of health and good social relationships and the non-material NCP of learning in the last period (Fig 6). The increases in the number of components and in network density (i.e., number of edges) could have resulted only from the increasing number of publications over time (Fig 2). The simulation of random datasets and associated networks showed that this was true for the number of components but not for network density (S4 Text). The increase in the number of components resulted from the increasing number of publications over time, as more publications were likely to cover a larger number of topics. But the high network density in the last time period could be not explained only by the large number of papers (S4 Text). In other terms, the most recent papers considered more diverse combinations of topics.

An increasing number of publications have assessed the interactive effects of different indirect drivers of change on fostering land-use change and climate change, as well as, the impact of these direct drivers on different NCP, namely food and feed, materials, climate regulation, soil formation, regulation of freshwater quality and quantity, habitat maintenance and physical experiences, such as recreation and aesthetic enjoyment (Fig 6). Similarly, the interlinkages between NCP and human wellbeing have been increasingly researched since 2011. In the last period (2014–2016), numerous publications have addressed the interlinkages between material and regulating NCP on basic materials, security, and health (Fig 6). By contrast, interlinkages between biodiversity and NCP received less attention in this period (Fig 6).

The IPBES components with highest weighted degree and betweenness also changed over time, although the impact of land-use change on biodiversity was consistently represented across the four periods of time. Until 2007, the regulating NCP of freshwater quality regulation, habitat maintenance and regulation of natural hazards, together with the non-material NCP of physical experiences and the pressure of land-use change were the components with the highest weighted degree and betweenness (Fig 6A; S2 Table). These regulating and non-material NCP, as well as land-use change, were the main focus of ecosystem service research in mountains and were also critical in connecting with other components of the IPBES framework. In the period 2008–2010, only the components of land-use change (direct driver of change) and the regulating NCP of soil formation presented high weighted degree and betweenness (Fig 6B; S3 Table). In 2011–2013, two material NCP (food and feed, and materials) together with regulation of climate presented the highest weighted degree and betweenness (Fig 6C; S4 Table). In this third period, the institutional response represented by conservation policies also appeared among the most important components (S4 Table). In the last period (2014–2016), components with the highest weighted degree were again the regulating NCP of freshwater quality and the direct driver of land-use change. However, new components also appeared as important, such as the NCP of physical experiences in nature, conservation responses, the indirect driver of changes in markets, and two components of wellbeing (i.e. basic material for a good life and security) (Fig 6D; S5 Table). In this period, the element with the highest betweenness was the non-material NCP of learning (S5 Table).

## 4. Discussion

This review presents the status of ecosystem service science in mountains by applying the IPBES conceptual framework as an analytical lens. In doing so, we identified major research gaps that should be addressed to provide interdisciplinary understanding of the status and trends of beneficial NCP in mountains. Addressing these gaps would contribute to future IPBES assessments [24] by providing an integrated understanding of the interlinkages between NCP, biodiversity, people’s quality of life, drivers of change and institutional responses. Current knowledge gaps found in this study include: (1) the interlinkages between biodiversity and NCP and between NCP and people’s quality of life (arrows 1 and 2 of Fig 1); (2) the effect of indirect drivers of change on direct drivers and on NCP (arrows 5 and 7 of Fig 1); (3) particular geographical areas, such as the Andes, the South draining parts of the Himalayas and Central Asian mountain ranges, the Ural Mountains and the Armenian and the Anatolian Plateaus (Fig 3); and (4) particular aspects of the IPBES components, specifically functional diversity, non-material NCP, the wellbeing dimensions of social relationships, health and freedom of choice, and the effect of demographic and cultural changes on NCP (Fig 5).

Some methodological limitations should be noted. First, this systematic review might overlook some of the research on specific ecosystem services in mountains because there may be publications that study ecosystem services without referring to them as ecosystem service, environmental service, ecosystem good or ecosystem function (see S1 Text). However, the aim of this study was to determine to what extent ecosystem service science in mountains has explored the different components of the IPBES conceptual framework, and therefore our search deliberately targeted those publications that had used the ecosystem service framework. Second, although we used the IPBES conceptual framework, we did not research detrimental NCP in mountains. Although detrimental NCP (or ecosystem disservices) have been increasingly studied in the scientific literature, research on this topic is still relatively marginal and is mainly focused on urban and agricultural systems and on biological invasions [56–59]. Therefore, future research is needed on detrimental NCP in mountains. Third, this study synthesizes the existing literature on ecosystem services in mountains and summarizes the interlinkages between the main components of the IPBES conceptual framework (Fig 1); however we do not provide the magnitude of the effect of one component over others and we do not analyze what factors trigger or jeopardize the provision of NCP. Although such information would be vital for decision-making, the results of our systematic review can still be useful for identifying what components of the IPBES conceptual framework need to be further researched in order to support future IPBES and national assessments. In addition, the results provided in this study can also contribute towards future agendas for mountain research by targeting those components that have been overlooked so far. Fourth, the search strategy used in this study allows the evaluation of the entire spectrum of research on ecosystem services in mountains up to 2016 (see S3 Text for the trends in 2017–2018), rather than focusing mainly on influential and top cited publications such as previous reviews on ecosystem service research [60–62]. However, as the search was done in English and dismissed gray literature in order to ensure the quality of studies included in the systematic review [63], an inherent bias may exist toward studies from countries with more capacity for publishing in international English-language journals. In fact, most of the studies on ecosystem services provided by mountains were conducted in Europe (Fig 3), a pattern that is consistent with previous systematic reviews on ecosystem services [18, 62,64,65]. Therefore, future systematic reviews on ecosystem service research need to recognize and include the knowledge built by researchers across Africa, South America and Asia [66]. In this sense, the recently published IPBES regional assessments represent an excellent example of including the knowledge around biodiversity and ecosystem services published in multiple languages and different formats, including gray literature [26–29].

### 4.1. Ecosystem service research in mountains over time

The temporal trend of ecosystem service research in mountains indicates that the number of scientific publications has increased over time until 2013, when the number of publications per year stabilized (Fig 2). Yet, ecosystem service research in mountains has led to fewer publications than in other ecosystems, such as agroecosystems [67] or urban systems [45]. The underrepresentation of mountains in ecosystem service research relative to other systems is consistent with other related fields, such as conservation biology [68] or ecology [69].

Alongside the increasing number of publications, research on ecosystem services in mountains has gradually studied more interlinkages between regulating and non-material NCP, dimensions of quality of life, and direct and indirect drivers of change (Fig 6). Likewise, methodological approaches have moved towards plural and integrated methodological approaches that required different disciplines (Fig 2). This result is consistent with the current pattern of ecosystem service research which has become more interdisciplinary [60,70] as a result of recent calls for integrating diverse values of NCP [71–73] and for including knowledge from social sciences and humanities [74–76], particularly in IPBES assessments [34,77–79]. Despite this recent recognition of the relevance of social sciences and humanities, socio-cultural methodological approaches are still less applied than biophysical and economic approaches in mountain research (Fig 2). This result is also consistent with previous systematic reviews on ecosystem services provided by agroecosystems [46], marine and coastal systems [47] and urban systems [44,45].

The abovementioned temporal evolution of research on ecosystem services in mountains aligns with the evolution of topics identified by Droste et al. [70] in ecosystem services, despite some differences in the temporal periods used for analysis. Droste et al. [70] found that ecosystem service research was founded on natural science with a focus on nature conservation and that economic valuation also played a major part between 1990 and 2010. In this research, we found that biophysical approaches focusing on biodiversity and regulating NCP as well as on the impact of direct drivers of change on biodiversity were dominant in the first period (1997–2007) of ecosystem service research in mountains (Figs 2 and 4), but we also found that economic valuation played an important role in this period (Fig 2). This result contradicts previous reviews of ecosystem service research that claimed the sole dominance of economic approaches in the period of 1990–2000 [60,80]. Similar to the findings of Chaudhary et al. [60] and Droste et al. [70], we found that in the last two periods of time (i.e. 2011–2013 and 2014–2016), ecosystem service research in mountains seeks to enhance the understanding of social-ecological systems by increasing the research on social aspects, such as people’s quality of life and indirect drivers of change (Figs 4 and 6). In these periods, we also observed that ecosystem service research became more policy-oriented by focusing more on the institutional responses associated with protected areas and market-based schemes, such as REDD+ (Reducing Emissions from Deforestation and Forest Degradation) programs and payments for ecosystem services (Figs 4 and 6).

### 4.2. Components of the IPBES conceptual framework studied in ecosystem service research in mountains

Besides the component on NCP (considered by all studies), the component of the IPBES conceptual framework on direct drivers of change was the second most frequently considered (77.5% of publications) followed by indirect drivers of change (45.0%). Yet, while research on direct drivers has decreased over time, research focusing on indirect drivers has increased (Fig 4). This result is consistent with global trends of science and recent findings of the IPBES regional assessments that show robust evidence regarding the impact of direct drivers of change, particularly land-use and climate change, on biodiversity and NCP [23,26,28,29,81]. By contrast, the effect of indirect drivers of change still remains as an important knowledge gap, in particular the effect of institutional responses on NCP (Fig 5). The fact that land-use change was by far the most studied direct driver (Figs 5B and 6) can be explained because it is the most important pressure affecting biodiversity and NCP [28,29,81]. In ecosystem service research, climate change has received less scientific attention than land-use change despite the fact that its impact on NCP is rapidly increasing worldwide [26–29], particularly in mountains [16,82]. However, this result may be misleading because the climate change scientific community does not often use the framework of ecosystem services [83] even though researchers are studying the impacts of climate change on biodiversity and ecosystem functioning (e.g. [84–86]). In addition, possible synergistic effects of land-use change and climate change on NCP in mountains [87–89], in both the present and future, are underexplored [88,90].

Regarding indirect drivers of change, policies and markets are the most prominent in ecosystem service research in mountains, followed by cultural change and agricultural policies (Figs 5B and 6). This result also supports the findings from the IPBES regional assessments that concluded that patterns of economic growth and trade as well as ineffective and inappropriate policies have accelerated the loss of biodiversity and NCP [26–29]. The IPBES regional assessments also identified population growth and urbanization as relevant drivers causing biodiversity decline [26–29]. By contrast, in mountains, rural abandonment has been increasingly considered as an indirect driver (Fig 6). In summary, indirect drivers associated with demographic changes, changes on cultural values and policy changes that underpin societal behavior are little understood and overall require more in-depth research in mountains [91,92].

Regarding the component of nature in the IPBES conceptual framework, this systematic review shows that the study of biodiversity in ecosystem service research in mountains has decreased over time (Fig 4). Although biodiversity is critical for the provision of NCP [24,93,94], much uncertainty remains over the interlinkages between biodiversity components and the different categories of NCP [25,95]. In particular, the biodiversity component of functional diversity is the least studied in mountains (Fig 5A) despite its relevance for the provision of NCP [96,97]. This result is consistent with the strong bias toward agroecosystems and forests found by Hevia et al. [45] in a systematic review on the links between direct drivers of change and ecosystem services via functional traits. The recent emergence of functional diversity and modelling and mapping approaches can contribute to enhancing knowledge about biodiversity-ecosystem services linkages and the synergies and trade-offs between biodiversity and ecosystem services in mountains (e.g. [98–100]).

The decreasing trend of research on biodiversity-ecosystem services linkages might be associated with the declining trend of research on regulating NCP (Fig 4). Nevertheless, regulating NCP were the most researched in mountains (Fig 5A), particularly the categories of regulation of climate, regulation of freshwater quality and quantity, soil formation and habitat maintenance (Fig 6; S2 Fig). Research on material and non-material NCP slightly increased over time (Fig 4), particularly the categories of food and feed, materials, physical experiences and supporting identities (Fig 6; S2 Fig). Nevertheless, non-material NCP are the category less studied in mountains (Fig 5A) despite the claim that mountains are hotspots of scenic beauty, recreation and spiritual enjoyment [9,100]. The fact that non-material NCP remained underexplored in ecosystem service research in mountains can explain the significant shortcomings in understanding the contributions of nature to people’s quality of life, particularly the dimensions of social relationships and freedom of choice (Fig 5A).

Only 34.3% of the studies connect NCP with people’s quality of life, particularly with material needs (e.g. [82,100,101]) (Fig 5). This result supports the findings from Grêt-Regamey et al. [3], showing that only 20% of publications on ecosystem services in mountains connected ecosystem functions with human wellbeing. In addition, research linking NCP with quality of life is framed in a simplistic way, mainly looking at the interactions between regulating and material NCP with basic materials or the effect of land-use change and climate change on livelihoods security (e.g. [82,102]) (Fig 6).

### 4.3. Moving towards policy-oriented research on ecosystem services in mountains

Ecosystem service research in mountains appears to have focused less on the institutional responses than other components of the IPBES conceptual framework (Fig 4), although it has become more policy- and action-oriented over time. The institutional responses assessed in the literature mainly focus on market-based schemes (Fig 5A). Indeed, REDD+ programs and payments for ecosystem services have increasingly been explored as management options in mountains across Latin America (e.g. [101,103,104]), Asia (e.g. [105,106]) and Africa (e.g. [107–109]). The programs of payments for ecosystem services assessed in the scientific literature on mountains seek to secure water, reduce deforestation and conserve local ecosystem services while enhancing the livelihoods of communities and eradicating poverty. By contrast, the reviewed literature on protected areas as institutional responses assessed changes in NCP in response to different conservation policies (e.g. [110,111]). In addition to these two institutional responses, NCP in mountains are also managed by informal institutions, such as community-based management. Community-based management is particularly relevant in the case of material NCP in forests (e.g. fuelwood, timber, and edible and medicinal plants) (e.g. [112–114]), supporting identities (e.g. sacred ceremonies, sense of place) (e.g. [112,115,116]) and regulation of freshwater quantity and quality (e.g. [117–119]). Research on community-based management in mountains focuses on sustaining the flow of NCP while simultaneously improving livelihoods, securing food, water and energy and fostering social relationships. The fact that community-based management remains underexplored may be explained by the few published analyses of social relationships as dimension of quality of life (Fig 5A). In-depth research on effective governance systems of ecosystem services in mountains is needed to find effective management actions that guarantee the sustainable flow of NCP while fulfilling the needs of different social actors and reducing poverty. Therefore, addressing this knowledge gap is essential to foster robust governance models that support sustainable management of NCP in mountains.

## Supporting information

S1 Table PRISMA checklist applied to the systematic review of ecosystem service research in mountains. (PDF)

S2 Table Important IPBES components emerging from the studies published on ecosystem service research in mountains until 2007. (PDF)

S3 Table Important IPBES components emerging from the studies published on ecosystem service research in mountains between 2008 and 2010. (PDF)

S4 Table Important IPBES components emerging from the studies published on ecosystem service research in mountains between 2011 and 2013. (PDF)

S5 Table Important IPBES components emerging from the studies published on ecosystem service research in mountains between 2014 and 2016. (PDF)

S1 Fig Flow diagram of the selection process of the articles used in this systematic review. (TIF)

S2 Fig Number of papers assessing each category of nature’s contributions to people, classified in material (blue), regulating (green) and non-material (teal). (TIF)

S1 Text Protocol of systematic review. (PDF)

S2 Text List of papers considered in the review. (PDF)

S3 Text Trends of research on nature’s contributions to people in mountains in the time period of 2017–2018 based on a random sample of 10% of the screened papers (*n* = 310). (PDF)

S4 Text Factoring out the effect of the number of papers on network indicators. (PDF)
