# Supplementary material for: Nature’s contributions to people in mountains: A review
Source: PLoS One. 2019 Jun 11;14(6):e0217847. doi: 10.1371/journal.pone.0217847 (PMC6559649; doi:10.1371/journal.pone.0217847)
Supplement: S2 Table — (PDF) [file pone.0217847.s002.pdf]

S2 Table. Important IPBES components emerging from the studies published on ecosystem service research in mountains until 2007.

	Element	IPBES	Weighted Degree	Betweenness
1	Reg. freshwater quality	Nature’s contributions to people	78	117.874
2	Physical experiences	Nature’s contributions to people	78	67.992
3	Land-use change	Direct driver	75	65.002
4	Habitat maintenance	Nature’s contributions to people	67	107.580
5	Food and feed	Nature’s contributions to people	66	18.496
6	Reg. hazards	Nature’s contributions to people	63	68.929
7	Soil formation	Nature’s contributions to people	48	27.839
8	Climate change	Direct driver	44	19.803
9	Markets	Indirect driver	43	9.020
10	Supporting identities	Nature’s contributions to people	41	6.262
