# Supplementary material for: Nature’s contributions to people in mountains: A review
Source: PLoS One. 2019 Jun 11;14(6):e0217847. doi: 10.1371/journal.pone.0217847 (PMC6559649; doi:10.1371/journal.pone.0217847)
Supplement: S3 Table — (PDF) [file pone.0217847.s003.pdf]

S3 Table. Important IPBES components emerging from the studies published on ecosystem service research in mountains between 2008 and 2010.

	Elements	IPBES	Weighted Degree	Betweenness
1	Land-use change	Direct driver	77	80.731
2	Soil formation	Nature’s contributions to people	68	50.038
3	Climate reg.	Nature’s contributions to people	67	22.231
4	Food and feed	Human wellbeing	56	17.334
5	Reg. freshwater quality	Nature’s contributions to people	48	12.649
6	Habitat maintenance	Nature’s contributions to people	45	11.322
7	Physical experiences	Nature’s contributions to people	45	8.771
8	Supporting identities	Nature’s contributions to people	37	1.343
9	Reg. freshwater quantity	Nature’s contributions to people	31	28.032
10	Community	Biodiversity	30	4.152
