# Supplementary material for: Nature’s contributions to people in mountains: A review
Source: PLoS One. 2019 Jun 11;14(6):e0217847. doi: 10.1371/journal.pone.0217847 (PMC6559649; doi:10.1371/journal.pone.0217847)
Supplement: S4 Table — (PDF) [file pone.0217847.s004.pdf]

S4 Table. Important IPBES components emerging from the studies published on ecosystem service research in mountains between 2011 and 2013.

	Elements	IPBES	Weighted Degree	Betweenness
1	Food and feed	Nature’s contributions to people	237	138.620
2	Climate reg.	Nature’s contributions to people	212	121.807
3	Materials	Nature’s contributions to people	183	66.727
4	Reg. freshwater quantity	Nature’s contributions to people	179	34.727
5	Habitat maintenance	Nature’s contributions to people	99	2.397
6	Land-use change	Direct driver	96	0.952
7	Reg. hazards	Nature’s contributions to people	88	6.208
8	Reg. air quality	Nature’s contributions to people	84	10.887
9	Conservation response	Institutional responses	84	1.202
10	Medicinal resources	Nature’s contributions to people	65	32.023
