# Supplementary material for: Nature’s contributions to people in mountains: A review
Source: PLoS One. 2019 Jun 11;14(6):e0217847. doi: 10.1371/journal.pone.0217847 (PMC6559649; doi:10.1371/journal.pone.0217847)
Supplement: S5 Table — (PDF) [file pone.0217847.s005.pdf]

S5 Table. Important IPBES components emerging from the studies published on ecosystem service research in mountains between 2014 and 2016.

	Elements	IPBES	Weighted Degree	Betweenness
1	Land-use change	Direct driver	320	6.494
2	Conservation response	Institutional responses	211	1.702
3	Physical experiences	Nature’s contributions to people	185	3.032
4	Reg. freshwater quality	Nature’s contributions to people	174	6.494
5	Basic material	Human wellbeing	147	2.307
6	Security	Human wellbeing	135	1.550
7	Conservation policy	Indirect driver	134	2.616
8	Markets	Indirect driver	130	3.032
9	Soil formation	Nature’s contributions to people	128	3.032
10	Learning	Nature’s contributions to people	76	33.663
