# Supplementary figures and images for: Nature’s contributions to people in mountains: A review
Source: PLoS One. 2019 Jun 11;14(6):e0217847. doi: 10.1371/journal.pone.0217847 (PMC6559649; doi:10.1371/journal.pone.0217847)

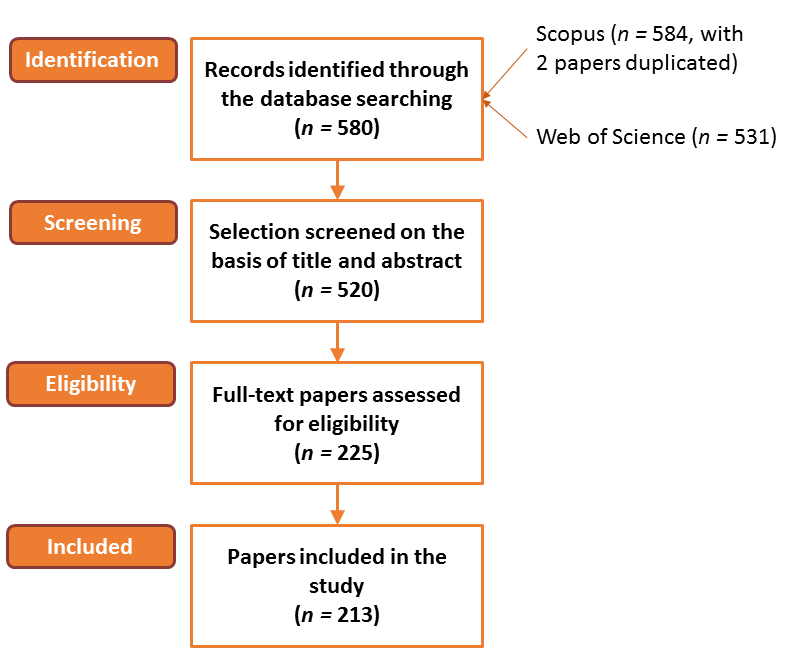

Supplement: S1 Fig — (TIF) [file pone.0217847.s006.tif]

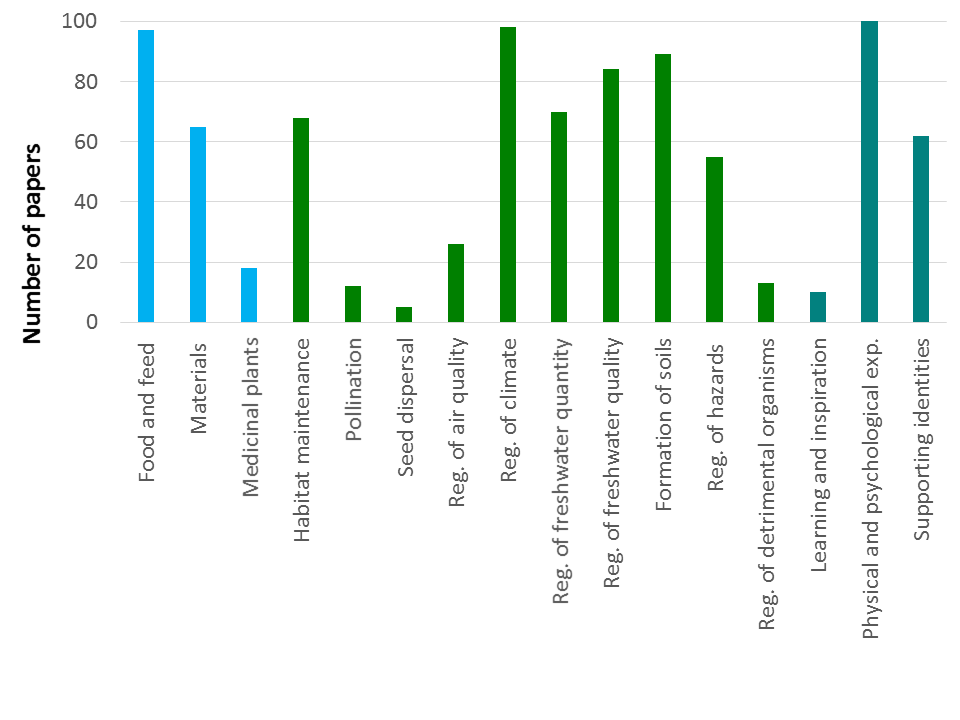

Supplement: S2 Fig — (TIF) [file pone.0217847.s007.tif]
