# Supplementary material for: Nature’s contributions to people in mountains: A review
Source: PLoS One. 2019 Jun 11;14(6):e0217847. doi: 10.1371/journal.pone.0217847 (PMC6559649; doi:10.1371/journal.pone.0217847)
Supplement: S1 Text — (PDF) [file pone.0217847.s008.pdf]

S1 Text. Protocol of systematic review

The terms searched in the title, abstract or keywords were:

1. Regarding mountains: (mountain* OR highland* OR paramo* OR cordiller* OR summit* OR andes OR andean OR alps OR alpine OR appalach* OR apennin* OR carpath* OR himalaya* OR karakoram* OR "Hindu Kush" OR ghats OR sierra OR serra OR macizo OR pamir OR tibet* OR "Tian Shan" OR kunlum OR ural OR kilimanjaro OR atlas)
2. Regarding assessment or valuation: (evalu* OR assess* OR valu* OR map* OR quantif* OR estimat*)
3. Regarding ecosystem services: ("ecosystem service*" OR "ecosystem good*" OR "ecosystem function*" OR "environmental service*" OR "environmental good*")
