# Supplementary material for: Nature’s contributions to people in mountains: A review
Source: PLoS One. 2019 Jun 11;14(6):e0217847. doi: 10.1371/journal.pone.0217847 (PMC6559649; doi:10.1371/journal.pone.0217847)
Supplement: S2 Text — (PDF) [file pone.0217847.s009.pdf]

S2 Text. List of papers considered in the review

Period 1997-2007

1. Higgins SI, Turpie JK, Costanza R, Cowling RM, Le Maitre DC, Marais C, et al. An ecological economic simulation model of mountain fynbos ecosystems dynamics, valuation and management. Ecol Econ. 1997;22: 155–169. doi:10.1016/S0921-8009(97)00575-2

2. Xue D, Tisdell C. Valuing ecological functions of biodiversity in\rChangbaishan Mountain Biosphere Reserve\rin Northeast China. Biodivers Conserv. 2001;10: 467–481.

3. Wu G, Xiao H, Zhao J, Shao G, Li J. Forest ecosystem services of changbai mountain in china. Sci China C Life Sci. 2002;45: 21–32. doi:10.1360/02yc9003

4. Peterson GD, Beard Jr. TD, Beisner BE, Bennett EM, Carpenter SR, Cumming GS, et al. Assessing future ecosystem services: a case study of the Northern Highlands Lake District, Wisconsin. Conserv Ecol. 2003;7: 1.

5. Farley KA, Kelly EF, Hofstede RGM. Soil Organic Carbon and Water Retention after Conversion of Grasslands to Pine Plantations in the Ecuadorian Andes. Ecosystems. 2004;7: 729–739. doi:10.1007/s10021-004-0047-5

6. Romero H, Ordenes F. Emerging Urbanization in the Southern Andes. Mt Res Dev. 2004;24: 197–201.

7. Semwal RL, Nautiyal S, Sen KK, Rana U, Maikhuri RK, Rao KS, et al. Patterns and ecological implications of agricultural land-use changes: A case study from central Himalaya, India. Agric Ecosyst Environ. 2004;102: 81–92. doi:10.1016/S0167-8809(03)00228-7

8. Pereira E, Queirós C, Pereira H, Vicente L. Ecosystem Services and Human Well – Being : A participatory study in a mountain community in Northern Portugal. Ecol Soc. 2005;10: 1–26.

9. Schröter D, Cramer W, Leemans R, Prentice IC, Araújo MB, Arnell NW, et al. Ecosystem service supply and vulnerability to global change in Europe. Science. 2005;310: 1333–7. doi:10.1126/science.1115233

10. Li J, Ren Z, Zhou Z. Ecosystem services and their values: A case study in the Qinba mountains of China. Ecol Res. 2006;21: 597–604. doi:10.1007/s11284-006-0148-z

11. Pederson G, Gray ST, Fagre DB, Graumlich LJ. Long-Duration Drought Variability and Impacts on Ecosystem Services: A Case Study from Glacier National Park, Montana. Earth Interact. 2006;10: 1-28.

12. Li RQ, Dong M, Cui JY, Zhang LL, Cui QG, He WM. Quantification of the impact of land-use changes on ecosystem services: A case study in Pingbian County, China. Environ Monit Assess. 2007;128: 503–510. doi:10.1007/s10661-006-9344-0

13. Quétier F, Lavorel S, Thuiller W, Davies I. Plant-trait-based modeling assessment of ecosystem service sensitivity to land-use change. Ecol Appl. 2007;17: 2377–2386. doi:10.1890/06-0750.1

14. Rammig A, Fahse L, Bebi P, Bugmann H. Wind disturbance in mountain forests: Simulating the impact of management strategies, seed supply, and ungulate browsing on forest succession. For Ecol Manage. 2007;242: 142–154. doi:10.1016/j.foreco.2007.01.036

15. Walz A, Lardelli C, Behrendt H, Grêt-Regamey A, Lundström C, Kytzia S, et al. Participatory scenario analysis for integrated regional modelling. Landsc Urban Plan. 2007;81: 114–131. doi:10.1016/j.landurbplan.2006.11.001

Period 2008-2010

16. Albert CH, Thuiller W, Lavorel S, Davies ID, Garbolino E. Land-use change and subalpine tree dynamics: colonization of *Larix decidua* in French subalpine grasslands. J Appl Ecol. Wiley/Blackwell (10.1111); 2008;45: 659–669. doi:10.1111/j.1365-2664.2007.01416.x

17. Grau HR, Hernández ME, Gutierrez J, Gasparri NI, Casavecchia MC, Flores-Ivaldi EE, et al. A Peri-Urban Neotropical Forest Transition and its Consequences for Environmental Services. Ecol Soc.; 2008;13: art35. doi:10.5751/ES-02434-130135

18. Grêt-Regamey A, Bebi P, Bishop ID, Schmid WA. Linking GIS-based models to value ecosystem services in an Alpine region. J Environ Manage.; 2008;89: 197–208. doi:10.1016/J.JENVMAN.2007.05.019

19. Grêt-Regamey A, Walz A, Bebi P. Valuing Ecosystem Services for Sustainable Landscape Planning in Alpine Regions. Mt Res Dev. International Mountain Society and United Nations University ; 2008;28: 156–165. doi:10.1659/mrd.0951

20. Heckmann KE, Manley PN, Schlesinger MD. Ecological integrity of remnant montane forests along an urban gradient in the Sierra Nevada. For Ecol Manage.; 2008;255: 2453–2466. doi:10.1016/J.FORECO.2008.01.005

21. Immerzeel W, Stoorvogel J, Antle J. Can payments for ecosystem services secure the water tower of Tibet? Agric Syst.; 2008;96: 52–63. doi:10.1016/J.AGSY.2007.05.005

22. Kellermann JL, Johnson MD, Stercho AM, Hackett SC. Ecological and economic services provided by birds on Jamaican Blue Mountain coffee farms. Conserv Biol. 2008;22: 1177–1185. doi:10.1111/j.1523-1739.2008.00968.x

23. Burt JW, Rice KJ. Not all ski slopes are created equal: Disturbance intensity affects ecosystem properties. Ecol Appl.; 2009;19: 2242–2253. doi:10.1890/08-0719.1

24. Carvajal A, Feijoo A, Quintero H, Rondón MA. Soil Organic Carbon in Different Land Uses of Colombian Andean Landscapes. Rev la Cienc del Suelo y Nutr Veg. Sociedad Chilena de la Ciencia del Suelo; 2009;December: 222–235.

25. Currie B, Milton SJ, Steenkamp JC. Cost–benefit analysis of alien vegetation clearing for water yield and tourism in a mountain catchment in the Western Cape of South Africa. Ecol Econ.; 2009;68: 2574–2579. doi:10.1016/J.ECOLECON.2009.04.007

26. Endreny TA, Gokcekus H. Ancient eco-technology of qanats for engineering a sustainable water supply in the Mediterranean Island of Cyprus. Environ Geol.; 2009;57: 249–257. doi:10.1007/s00254-008-1274-4

27. Fries A, Rollenbeck R, Göttlicher D, Nauss T, Homeier J, Peters T, et al. Thermal Structure of a Megadiverse Andean Mountain Ecosystem in Southern Ecuador and Its Regionalization. Erdkunde; 2009;63: 321–335. doi:10.2307/25648253

28. Hadgu KM, Kooistra L, Rossing WAH, van Bruggen AHC. Assessing the effect of Faidherbia albida based land use systems on barley yield at field and regional scale in the highlands of Tigray, Northern Ethiopia. Food Secur.; 2009;1: 337–350. doi:10.1007/s12571-009-0030-2

29. Lara A, Little C, Urrutia R, McPhee J, Álvarez-Garretón C, Oyarzún C, et al. Assessment of ecosystem services as an opportunity for the conservation and management of native forests in Chile. For Ecol Manage.; 2009;258: 415–424. doi:10.1016/J.FORECO.2009.01.004

30. Loheide SP, Lundquist JD. Snowmelt-induced diel fluxes through the hyporheic zone. Water Resour Res.; 2009;45. doi:10.1029/2008WR007329

31. Martínez ML, Pérez-Maqueo O, Vázquez G, Castillo-Campos G, García-Franco J, Mehltreter K, et al. Effects of land use change on biodiversity and ecosystem services in tropical montane cloud forests of Mexico. For Ecol Manage.; 2009;258: 1856–1863. doi:10.1016/J.FORECO.2009.02.023

32. Miralles I, Ortega R, Almendros G, Sánchez-Marañón M, Soriano M. Soil quality and organic carbon ratios in mountain agroecosystems of South-east Spain. Geoderma.; 2009;150: 120–128. doi:10.1016/J.GEODERMA.2009.01.011

33. Naidoo R, Malcolm T, Tomasek A. Economic benefits of standing forests in highland areas of Borneo: quantification and policy impacts. Conserv Lett. ; 2009;2: 36–45. doi:10.1111/j.1755-263X.2008.00041.x

34. Pauchard A, Kueffer C, Dietz H, Daehler CC, Alexander J, Edwards PJ, et al. Ain’t no mountain high enough: plant invasions reaching new elevations. Front Ecol Environ.; 2009;7: 479–486. doi:10.1890/080072

35. Quintero M, Wunder S, Estrada RD. For services rendered? Modeling hydrology and livelihoods in Andean payments for environmental services schemes. For Ecol Manage.; 2009;258: 1871–1880. doi:10.1016/J.FORECO.2009.04.032

36. Schroth G, Laderach P, Dempewolf J, Philpott S, Haggar J, Eakin H, et al. Towards a climate change adaptation strategy for coffee communities and ecosystems in the Sierra Madre de Chiapas, Mexico. Mitig Adapt Strateg Glob Chang; 2009;14: 605–625. doi:10.1007/s11027-009-9186-5

37. Teich M, Bebi P. Evaluating the benefit of avalanche protection forest with GIS-based risk analyses—A case study in Switzerland. For Ecol Manage.; 2009;257: 1910–1919. doi:10.1016/J.FORECO.2009.01.046

38. Tenhunen J, Geyer R, Adiku S, Reichstein M, Tappeiner U, Bahn M, et al. Influences of changing land use and CO2 concentration on ecosystem and landscape level carbon and water balances in mountainous terrain of the Stubai Valley, Austria. Glob Planet Change.; 2009;67: 29–43. doi:10.1016/J.GLOPLACHA.2008.12.010

39. Townsend PA, Helmers DP, Kingdon CC, McNeil BE, de Beurs KM, Eshleman KN. Changes in the extent of surface mining and reclamation in the Central Appalachians detected using a 1976–2006 Landsat time series. Remote Sens Environ.; 2009;113: 62–

72. doi:10.1016/J.RSE.2008.08.012

40. Biervliet O van, Wiśniewski K, Daniels J, Vonesh JR. Effects of Tea Plantations on Stream Invertebrates in a Global Biodiversity Hotspot in Africa. Biotropica. Association for Tropical Biology and Conservation; 2009;41: 469–475. doi:10.2307/27742800

41. Wang C, Meer P van der, Peng M, Douven W, Hessel R, Dang C. Ecosystem Services Assessment of Two Watersheds of Lancang River in Yunnan, China with a Decision Tree Approach. AMBIO A J Hum Environ.; 2009;38: 47–54. doi:10.1579/0044-7447-38.1.47

42. Blignaut J, Mander M, Schulze R, Horan M, Dickens C, Pringle C, et al. Restoring and managing natural capital towards fostering economic development: Evidence from the Drakensberg, South Africa. Ecol Econ; 2010;69: 1313–1323. doi:10.1016/J.ECOLECON.2010.01.007

43. Descheemaeker K, Mapedza E, Amede T, Ayalneh W. Effects of integrated watershed management on livestock water productivity in water scarce areas in Ethiopia. Phys Chem Earth, Parts A/B/C.; 2010;35: 723–729. doi:10.1016/J.PCE.2010.06.006

44. Kijazi MH, Kant S. Forest stakeholders' value preferences in Mount Kilimanjaro, Tanzania. For Policy Econ.; 2010;12: 357–369. doi:10.1016/J.FORPOL.2010.02.007

45. Li J, Wang W, Hu G, Wei Z. Changes in ecosystem service values in Zoige Plateau, China. Agric Ecosyst Environ.; 2010;139: 766–770. doi:10.1016/J.AGEE.2010.10.019

46. Null SE, Viers JH, Mount JF. Hydrologic Response and Watershed Sensitivity to Climate Warming in California's Sierra Nevada. PLoS One.; 2010;5: e9932. doi:10.1371/journal.pone.0009932

47. Quétier F, Rivoal F, Marty P, Chazal J, Thuiller W, Lavorel S. Social representations of an alpine grassland landscape and socio-political discourses on rural development. Reg Environ Chang. 2009;10: 119–130. doi:10.1007/s10113-009-0099-3

48. Schwandt JW, Lockman IB, Kliejunas JT, Muir JA. Current health issues and management strategies for white pines in the western United States and Canada. For Pathol.; 2010;40: 226–250. doi:10.1111/j.1439-0329.2010.00656.x

Period 2011-2013

49. Buytaert W, Cuesta-Camacho F, Tobón C. Potential impacts of climate change on the environmental services of humid tropical alpine regions. Glob Ecol Biogeogr.; 2011;20: 19–33. doi:10.1111/j.1466-8238.2010.00585.x

50. Castro AJ, Martín-López B, García-LLorente M, Aguilera PA, López E, Cabello J. Social preferences regarding the delivery of ecosystem services in a semiarid Mediterranean region. J Arid Environ. 2011;75: 1201–1208. doi:10.1016/j.jaridenv.2011.05.013

51. Dong X, Zhang Y, Cui W, Xun B, Yu B, Ulgiati S, et al. Emergy-Based Adjustment of the Agricultural Structure in a Low-Carbon Economy in Manas County of China. Energies. Molecular Diversity Preservation International; 2011;4: 1428–1442.

doi:10.3390/en4091428

52. Fisher B, Turner RK, Burgess ND, Swetnam RD, Green J, Green RE, et al. Measuring, modeling and mapping ecosystem services in the Eastern Arc Mountains of Tanzania. Prog Phys Geogr. 2011;35: 595–611. doi:10.1177/0309133311422968

53. Lamarque P, Tappeiner U, Turner C, Steinbacher M, Bardgett RD, Szukics U, et al. Stakeholder perceptions of grassland ecosystem services in relation to knowledge on soil fertility and biodiversity. Reg Environ Chang.; 2011;11: 791–804. doi:10.1007/s10113-011-0214-0

54. Lavorel S, Grigulis K, Lamarque P, Colace M-P, Garden D, Girel J, et al. Using plant functional traits to understand the landscape distribution of multiple ecosystem services. J Ecol.; 2011;99: 135–147. doi:10.1111/j.1365-2745.2010.01753.x

55. Mekuria W, Veldkamp E, Tilahun M, Olschewski R. Economic Valuation of Land Restoration: the Case of Exclosures Established on Communal Grazing Lands in Tigray, Ethiopia. L Degrad Dev. 2011;22: 334–344. doi:10.1002/ldr.1001

56. Moore CC, Holmes TP, Bell KP. An attribute-based approach to contingent valuation of forest protection programs. J For Econ.; 2011;17: 35–52. doi:10.1016/J.JFE.2010.09.001

57. Narloch U, Pascual U, Drucker Ag. Cost-effectiveness targeting under multiple conservation goals and equity considerations in the Andes. Environ Conserv.; 2011;38: 417–425. doi:10.1017/S0376892911000397

58. Nie Y, Li A. Assessment of Alpine Wetland Dynamics from 1976–2006 in the Vicinity of Mount Everest. Wetlands.; 2011;31: 875–884. doi:10.1007/s13157-011-0202-7

59. Norton JB, Jungst LJ, Norton U, Olsen HR, Tate KW, Horwath WR. Soil Carbon and Nitrogen Storage in Upper Montane Riparian Meadows. Ecosystems.; 2011;14: 1217–1231. doi:10.1007/s10021-011-9477-z

60. Notaro S, Paletto A. Links between Mountain Communities and Environmental Services in the Italian Alps. Sociol Ruralis. 2011;51: 137–157. doi:10.1111/j.1467-9523.2011.00532.x

61. Stratford CJ, Acreman MC, Rees HG. A simple method for assessing the vulnerability of wetland ecosystem services. Hydrol Sci J.; 2011;56: 1485–1500. doi:10.1080/02626667.2011.630669

62. Swetnam RD, Fisher B, Mbilinyi BP, Munishi PKT, Willcock S, Ricketts T, et al. Mapping socio-economic scenarios of land cover change: A GIS method to enable ecosystem service modelling. J Environ Manage.; 2011;92: 563–574. doi:10.1016/J.JENVMAN.2010.09.007

63. Wardrop DH, Glasmeier AK, Peterson-Smith J, Eckles D, Ingram H, Brooks RP. Wetland ecosystem services and coupled socioeconomic benefits through conservation practices in the Appalachian Region. Ecol Appl.; 2011;21: S93–S115. doi:10.1890/09-2292.1

64. Zipper CE, Burger JA, Skousen JG, Angel PN, Barton CD, Davis V, et al. Restoring Forests and Associated Ecosystem Services on Appalachian Coal Surface Mines. Environ Manage. 2011;47: 751–765. doi:10.1007/s00267-011-9670-z

65. Krishna Bahadur KC. Spatio-temporal patterns of agricultural expansion and its effect on watershed degradation: a case from the mountains of Nepal. Environ Earth Sci. Springer-Verlag; 2012;65: 2063–2077. doi:10.1007/s12665-011-1186-6

66. Band LE, Hwang T, Hales TC, Vose J, Ford C. Ecosystem processes at the watershed scale: Mapping and modeling ecohydrological controls of landslides. Geomorphology.; 2012;137: 159–167. doi:10.1016/J.GEOMORPH.2011.06.025

67. Bowman WD, Murgel J, Blett T, Porter E. Nitrogen critical loads for alpine vegetation and soils in Rocky Mountain National Park. J Environ Manage.; 2012;103: 165–171. doi:10.1016/J.JENVMAN.2012.03.002

68. Briner S, Elkin C, Huber R, Grêt-Regamey A. Assessing the impacts of economic and climate changes on land-use in mountain regions: A spatial dynamic modeling approach. Agric Ecosyst Environ.; 2012;149: 50–63. doi:10.1016/J.AGEE.2011.12.011

69. Buytaert W, De Bièvre B. Water for cities: The impact of climate change and demographic growth in the tropical Andes. Water Resour Res.; 2012;48. doi:10.1029/2011WR011755

70. Carrer M, Motta R, Nola P. Significant Mean and Extreme Climate Sensitivity of Norway Spruce and Silver Fir at Mid-Elevation Mesic Sites in the Alps. Bernacchi CJ, editor. PLoS One.; 2012;7: e50755. doi:10.1371/journal.pone.0050755

71. Fries A, Rollenbeck R, Nauß T, Peters T, Bendix J. Near surface air humidity in a megadiverse Andean mountain ecosystem of southern Ecuador and its regionalization. Agric For Meteorol.; 2012;152: 17–30. doi:10.1016/J.AGRFORMET.2011.08.004

72. García-Llorente M, Martín-López B, Iniesta-Arandia I, López-Santiago CA, Aguilera PA, Montes C. The role of multi-functionality in social preferences toward semi-arid rural landscapes: An ecosystem service approach. Environ Sci Policy. 2012;19–20: 136–146. doi:10.1016/j.envsci.2012.01.006

73. Garrard R, Kohler T, Wiesmann U, Price MF, Byers AC, Sherp AR. Depicting community perspectives: repeat photography and participatory research as tools for assessing environmental services in Sagarmatha National Park, Nepal. Journal Prot Mt Areas Res. 2012;4: 21–31. doi:10.1553/eco.mont-4-2s21

74. Grêt-Regamey A, Brunner SH, Kienast F. Mountain Ecosystem Services: Who Cares? Mt Res Dev.; 2012;32: S23–S34. doi:10.1659/MRD-JOURNAL-D-10-00115.S1

75. Izquierdo AE, Clark ML. Spatial Analysis of Conservation Priorities Based on Ecosystem Services in the Atlantic Forest Region of Misiones, Argentina. Forests; 2012;3: 764–786. doi:10.3390/f3030764

76. Khan SM, Page S, Ahmad H, Harper D. Anthropogenic influences on the natural ecosystem of the naran valley in the western himalayas. Pakistan J Bot. 2012;44: 231–238.

77. Montagna M, Lozzia CG, Giorgi A, Baumgärtner J. Insect community structure and insect biodiversity conservation in an Alpine wetland subjected to an intermediate diversified management regime. Ecol Eng.; 2012;47: 242–246. doi:10.1016/J.ECOLENG.2012.06.025

78. Moreno-Sanchez R, Maldonado JH, Wunder S, Borda-Almanza C. Heterogeneous

users and willingness to pay in an ongoing payment for watershed protection initiative in the Colombian Andes. Ecol Econ.; 2012;75: 126–134. doi:10.1016/J.ECOLECON.2012.01.009

79. Notaro S, Paletto A. The economic valuation of natural hazards in mountain forests: An approach based on the replacement cost method. J For Econ.; 2012;18: 318–328. doi:10.1016/J.JFE.2012.06.002

80. Poveda K, Martínez E, Kersch-Becker MF, Bonilla MA, Tscharntke T. Landscape simplification and altitude affect biodiversity, herbivory and Andean potato yield. J Appl Ecol.; 2012;49: 513–522. doi:10.1111/j.1365-2664.2012.02120.x

81. Raboin ML, Posner JL. Pine or Pasture? Estimated Costs and Benefits of Land Use Change in the Peruvian Andes. Mt Res Dev.; 2012;32: 158–168. doi:10.1659/MRD-JOURNAL-D-10-00099.1

82. Schaafsma M, Morse-Jones S, Posen P, Swetnam RD, Balmford A, Bateman IJ, et al. Towards transferable functions for extraction of Non-timber Forest Products: A case study on charcoal production in Tanzania. Ecol Econ.; 2012;80: 48–62. doi:10.1016/J.ECOLECON.2012.04.026

83. Su S, Xiao R, Jiang Z, Zhang Y. Characterizing landscape pattern and ecosystem service value changes for urbanization impacts at an eco-regional scale. Appl Geogr.; 2012;34: 295–305. doi:10.1016/J.APGEOG.2011.12.001

84. Torres-Lezama A, Vilanova E, Ramírez-Angulo H, Alciaturi G. Socioeconomic and Environmental Basis for the Development of Small Scale Forestry in a Highly Degraded Watershed in the Venezuelan Andes. Small-scale For.; 2012;11: 321–337. doi:10.1007/s11842-011-9186-7

85. Álvarez S, Rubio A. Carbon baseline in a mixed pine-oak forest in the Juarez mountain range (Oaxaca, Mexico) using the co2fix v.3.2 model. Rev Chapingo Ser Ciencias For y del Ambient. 2013;XIX: 125–137. doi:10.5154/r.rchscfa.2012.01.005

86. Arndt N, Vacik H, Koch V, Arpaci A, Gossow H. Modeling human-caused forest fire ignition for assessing forest fire danger in Austria. IForest. 2013;6: 315–325. doi:10.3832/ifor0936-006

87. Bangash RF, Passuello A, Sanchez-Canales M, Terrado M, López A, Elorza FJ, et al. Ecosystem services in Mediterranean river basin: Climate change impact on water provisioning and erosion control. Sci Total Environ.; 2013;458–460: 246–255. doi:10.1016/J.SCITOTENV.2013.04.025

88. Bastian O. The role of biodiversity in supporting ecosystem services in Natura 2000 sites. Ecol Indic.; 2013;24: 12–22. doi:10.1016/J.ECOLIND.2012.05.016

89. Briner S, Elkin C, Huber R. Evaluating the relative impact of climate and economic changes on forest and agricultural ecosystem services in mountain regions. J Environ Manage.; 2013;129: 414–422. doi:10.1016/J.JENVMAN.2013.07.018

90. Briner S, Huber R, Bebi P, Elkin C, Schmatz DR, Grêt-Regamey A. Trade-Offs between Ecosystem Services in a Mountain Region. Ecol Soc.; 2013;18: art35. doi:10.5751/ES-05576-180335

91. Cai W, Yang J, Liu Z, Hu Y, Weisberg PJ. Post-fire tree recruitment of a boreal larch

forest in Northeast China. For Ecol Manage.; 2013;307: 20–29. doi:10.1016/J.FORECO.2013.06.056

92. Caruso BS, O'Sullivan AD, Faulkner S, Sherratt M, Clucas R. Agricultural Diffuse Nutrient Pollution Transport in a Mountain Wetland Complex. Water, Air, Soil Pollut.; 2013;224: 1695. doi:10.1007/s11270-013-1695-x

93. Elkin C, Gutiérrez AG, Leuzinger S, Manusch C, Temperli C, Rasche L, et al. A 2 °C warmer world is not safe for ecosystem services in the European Alps. Glob Chang Biol. 2013;19: 1827–1840. doi:10.1111/gcb.12156

94. Giday K, Eshete G, Barklund P, Aertsen W, Muys B. Wood biomass functions for Acacia abyssinica trees and shrubs and implications for provision of ecosystem services in a community managed exclosure in Tigray, Ethiopia. J Arid Environ.; 2013;94: 80–86. doi:10.1016/J.JARIDENV.2013.03.001

95. Grêt-Regamey A, Brunner SH, Altwegg J, Bebi P. Facing uncertainty in ecosystem services-based resource management. J Environ Manage.; 2013;127: S145–S154. doi:10.1016/J.JENVMAN.2012.07.028

96. Grêt-Regamey A, Brunner SH, Altwegg J, Christen M, Bebi P. Integrating Expert Knowledge into Mapping Ecosystem Services Trade-offs for Sustainable Forest Management. Ecol Soc.; 2013;18: art34. doi:10.5751/ES-05800-180334

97. Higuera D, Martín-López B, Sánchez-Jabba A. Social preferences towards ecosystem services provided by cloud forests in the neotropics: implications for conservation strategies. Reg Environ Chang. 2012;13: 861–872. doi:10.1007/s10113-012-0379-1

98. Hirschi C, Widmer A, Briner S, Huber R. Combining Policy Network and Model-Based Scenario Analyses: An Assessment of Future Ecosystem Goods and Services in Swiss Mountain Regions. Ecol Soc.; 2013;18: art42. doi:10.5751/ES-05480-180242

99. Huber R, Briner S, Peringer A, Lauber S, Seidl R, Widmer A, et al. Modeling Social-Ecological Feedback Effects in the Implementation of Payments for Environmental Services in Pasture-Woodlands. Ecol Soc.; 2013;18: art41. doi:10.5751/ES-05487-180241

100. Inauen N, Körner C, Hiltbrunner E. Hydrological consequences of declining land use and elevated CO2 in alpine grassland. J Ecol. 2013;101: 86–96. doi:10.1111/1365-2745.12029

101. Jindal R, Kerr JM, Ferraro PJ, Swallow BM. Social dimensions of procurement auctions for environmental service contracts: Evaluating tradeoffs between cost-effectiveness and participation by the poor in rural Tanzania. Land use policy.; 2013;31: 71–80. doi:10.1016/J.LANDUSEPOL.2011.11.008

102. Kaczan D, Swallow BM, Adamowicz WL (Vic). Designing a payments for ecosystem services (PES) program to reduce deforestation in Tanzania: An assessment of payment approaches. Ecol Econ; 2013;95: 20–30. doi:10.1016/J.ECOLECON.2013.07.011

103. Khan SM, Page S, Ahmad H, Shaheen H, Ullah Z, Ahmad M, et al. Medicinal flora and ethnoecological knowledge in the Naran Valley, Western Himalaya, Pakistan. J Ethnobiol Ethnomed.; 2013;9: 4. doi:10.1186/1746-4269-9-4

104. Khan SM, Page SE, Ahmad H, Harper DM. Sustainable utilization and conservation of

plant biodiversity in montane ecosystems: the western Himalayas as a case study. Ann Bot. 2013;112: 479–501. doi:10.1093/aob/mct125

105. Ko DW, Lee D. Dendroecological reconstruction of the disturbance dynamics and human legacy in an old-growth hardwood forest in Korea. For Ecol Manage.; 2013;302: 43–53. doi:10.1016/J.FORECO.2013.03.047

106. Li Y, Viña A, Yang W, Chen X, Zhang J, Ouyang Z, et al. Effects of conservation policies on forest cover change in giant panda habitat regions, China. Land use policy.; 2013;33: 42–53. doi:10.1016/J.LANDUSEPOL.2012.12.003

107. Lu C, Yu G, Xiao Y, Xie G. Wind tunnel simulation and evaluation of soil conservation function of alpine grassland in Qinghai–Tibet Plateau. Ecol Econ.; 2013;86: 16–20. doi:10.1016/J.ECOLECON.2012.10.015

108. Mendoza-Hernández PE, Orozco-Segovia A, Meave JA, Valverde T, Martínez-Ramos M. Vegetation recovery and plant facilitation in a human-disturbed lava field in a megacity: searching tools for ecosystem restoration. Plant Ecol.; 2013;214: 153–167. doi:10.1007/s11258-012-0153-y

109. Meyfroidt P. Environmental Cognitions, Land Change and Social-Ecological Feedbacks: Local Case Studies of Forest Transition in Vietnam. Hum Ecol.; 2013;41: 367–392. doi:10.1007/s10745-012-9560-x

110. Morán-Ordóñez A, Bugter R, Suárez-Seoane S, de Luis E, Calvo L. Temporal Changes in Socio-Ecological Systems and Their Impact on Ecosystem Services at Different Governance Scales: A Case Study of Heathlands. Ecosystems.; 2013;16: 765–782. doi:10.1007/s10021-013-9649-0

111. Moretti M, de Bello F, Ibanez S, Fontana S, Pezzatti GB, Dziock F, et al. Linking traits between plants and invertebrate herbivores to track functional effects of land-use changes. Pakeman R, editor. J Veg Sci.; 2013;24: 949–962. doi:10.1111/jvs.12022

112. Narloch U, Pascual U, Drucker AG. How to achieve fairness in payments for ecosystem services? Insights from agrobiodiversity conservation auctions. Land use policy.; 2013;35: 107–118. doi:10.1016/J.LANDUSEPOL.2013.05.002

113. Neudert R, Etzold J, Münzner F, Manthey M, Busse S. The Opportunity Costs of Conserving Pasture Resources for Mobile Pastoralists in the Greater Caucasus. Landsc Res. 2013;38: 499–522. doi:10.1080/01426397.2012.728204

114. Palomo I, Martín-López B, Potschin M, Haines-Young R, Montes C. National Parks , buffer zones and surrounding lands : Mapping ecosystem service flows. Ecosyst Serv. 2013;4: 104–116. doi:10.1016/j.ecoser.2012.09.001

115. Pop AI, Mihăiescu R, Mihăiescu T, Oprea MG, Tănăselia C, Ozunu A. Physico-chemical properties of some glacial lakes in the Romanian carpathians. Carpathian J Earth Environ Sci. 2013;8: 5–11.

116. Powers EM, Marshall JD, Zhang J, Wei L. Post-fire management regimes affect carbon sequestration and storage in a Sierra Nevada mixed conifer forest. For Ecol Manage.; 2013;291: 268–277. doi:10.1016/J.FORECO.2012.07.038

117. Qasim M, Hubacek K, Termansen M. Underlying and proximate driving causes of land use change in district Swat, Pakistan. Land use policy; 2013;34: 146–157.

doi:10.1016/J.LANDUSEPOL.2013.02.008

118. Ren GH, Deng B, Shang ZH, Hou Y, Long RJ. Plant communities and soil variations along a successional gradient in an alpine wetland on the Qinghai-Tibetan Plateau. Ecol Eng.; 2013;61: 110–116. doi:10.1016/J.ECOLENG.2013.09.017

119. Wen L, Dong S, Li Y, Li X, Shi J, Wang Y, et al. Effect of Degradation Intensity on Grassland Ecosystem Services in the Alpine Region of Qinghai-Tibetan Plateau, China. Schumann GJ-P, editor. PLoS One.; 2013;8: e58432. doi:10.1371/journal.pone.0058432

120. Yu P, Wang Y, Du A, Guan W, Feger K-H, Schwärzel K, et al. The effect of site conditions on flow after forestation in a dryland region of China. Agric For Meteorol.; 2013;178–179: 66–74. doi:10.1016/J.AGRFORMET.2013.02.007

Period 2014-2016

121. Duru M, Jouany C, Le Roux X, Navas ML, Cruz P. From a conceptual framework to an operational approach for managing grassland functional diversity to obtain targeted ecosystem services: Case studies from French mountains. Renewable Agriculture and Food Systems 2013; 29(3):1-16. doi:10.1017/S1742170513000306

122. Palomo I, Martín-López B, Alcorlo P, Montes C. Limitations of Protected Areas Zoning in Mediterranean Cultural Landscapes Under the Ecosystem Services Approach. Ecosystems. 2014;17: 1202–1215. doi:10.1007/s10021-014-9788-y

123. Sherrouse BC, Semmens DJ. Validating a method for transferring social values of ecosystem services between public lands in the Rocky Mountain region. Ecosyst Serv.; 2014;8: 166–177. doi:10.1016/J.ECOSER.2014.03.008

124. Khamis K, Hannah DM, Clarvis MH, Brown LE, Castella E, Milner AM. Alpine aquatic ecosystem conservation policy in a changing climate. Environ Sci Policy.; 2014;43: 39–55. doi:10.1016/J.ENVSCI.2013.10.004

125. Ferrari M, Geneletti D. Mapping and assessing multiple ecosystem services in an alpine region: a study in Trentino, Italy. Ann di Bot. 2014;4: 65–71. doi:10.4462/ANNBOTRM-11729

126. Pan Y, Wu J, Xu Z. Analysis of the tradeoffs between provisioning and regulating services from the perspective of varied share of net primary production in an alpine grassland ecosystem. Ecol Complex.; 2014;17: 79–86. doi:10.1016/J.ECOCOM.2013.11.001

127. Birch JC, Thapa I, Balmford A, Bradbury RB, Brown C, Butchart SHM, et al. What benefits do community forests provide, and to whom? A rapid assessment of ecosystem services from a Himalayan forest, Nepal. Ecosyst Serv.; 2014;8: 118–127. doi:10.1016/J.ECOSER.2014.03.005

128. Schirpke U, Tasser E, Tappeiner U. Mapping ecosystem services supply in mountain regions: a case study from South Tyrol (Italy). Ann di Bot. 2014;4: 35–43. doi:10.4462/ANNBOTRM-11599

129. Bremer LL, Farley KA, Lopez-Carr D, Romero J. Conservation and livelihood

outcomes of payment for ecosystem services in the Ecuadorian Andes: What is the potential for 'win–win'? Ecosyst Serv.; 2014;8: 148–165. doi:10.1016/J.ECOSER.2014.03.007

130. Woodhouse E, McGowan P, Milner-Gulland EJ. Fungal gold and firewood on the Tibetan plateau: examining access to diverse ecosystem provisioning services within a rural community. Oryx.; 2014;48: 30–38. doi:10.1017/S0030605312001330

131. Shi P, Yu D. Assessing urban environmental resources and services of Shenzhen, China: A landscape-based approach for urban planning and sustainability. Landsc Urban Plan. 2014;125: 290–297. doi:10.1016/J.LANDURBPLAN.2014.01.025

132. Notte A La, Scolozzi R, Molfetta P, Gubert F, Molignoni R, Franchi R, et al. An ecosystem service-based approach to design agri-environment-climate payments for the rural development programs 2014-2020. The case of the autonomous province of Trento. Ann di Bot. 2014;4: 91–96. doi:10.4462/ANNBOTRM-11646

133. Bayliss J, Schaafsma M, Balmford A, Burgess ND, Green JMH, Madoffe SS, et al. The current and future value of nature-based tourism in the Eastern Arc Mountains of Tanzania. Ecosyst Serv.; 2014;8: 75–83. doi:10.1016/J.ECOSER.2014.02.006

134. Entenmann SK, Schmitt CB, Konold W. REDD+-related activities in Kenya: actors' views on biodiversity and monitoring in a broader policy context. Biodivers Conserv.; 2014;23: 3561–3586. doi:10.1007/s10531-014-0821-4

135. Vásquez E, Ladd B, Borchard N. Carbon storage in a high-altitude Polylepis woodland in the Peruvian Andes. Alp Bot.; 2014;124: 71–75. doi:10.1007/s00035-014-0126-y

136. Bremer LL, Farley KA, Lopez-Carr D. What factors influence participation in payment for ecosystem services programs? An evaluation of Ecuador's SocioPáramo program. Land use policy.; 2014;36: 122–133. doi:10.1016/J.LANDUSEPOL.2013.08.002

137. Andrade Castañeda H, Espinoza Gómez E, Moreno Báltan H. Impact of grazing on soil organic storage carbon in high lands of Anaime, Tolima, Colombia. Zootec Trop. 2014;32: 7–21.

138. Trueman M, Standish RJ, Hobbs RJ. Identifying management options for modified vegetation: Application of the novel ecosystems framework to a case study in the Galapagos Islands. Biol Conserv.; 2014;172: 37–48. doi:10.1016/J.BIOCON.2014.02.005

139. Hegazy AK, Alatar AA, Thomas J, Faisal M, Alfarhan AH, Krzywinski K. Compatibility and complementarity of indigenous and scientific knowledge of wild plants in the highlands of southwest Saudi Arabia. J For Res.; 2014;25: 437–444. doi:10.1007/s11676-014-0473-y

140. Singh S, Mishra A. Deforestation-induced costs on the drinking water supplies of the Mumbai metropolitan, India. Glob Environ Chang.; 2014;27: 73–83. doi:10.1016/J.GLOENVCHA.2014.04.020

141. Liu XY, Liang TG, Guo ZG, Long RJ. A rangeland management pattern based on functional classification in the northern Tibetan region of China. L Degrad Dev.; 2014;25: 193–201. doi:10.1002/ldr.2139

142. Schaafsma M, Morse-Jones S, Posen P, Swetnam RD, Balmford A, Bateman IJ, et al.

The importance of local forest benefits: Economic valuation of Non-Timber Forest Products in the Eastern Arc Mountains in Tanzania. Glob Environ Chang.; 2014;24: 295–305. doi:10.1016/J.GLOENVCHA.2013.08.018

143. Cámara-Leret R, Paniagua-Zambrana N, Balslev H, Barfod A, Copete JC, Macía MJ. Ecological community traits and traditional knowledge shape palm ecosystem services in northwestern South America. For Ecol Manage.; 2014;334: 28–42. doi:10.1016/J.FORECO.2014.08.019

144. Ward A, Dargusch P, Thomas S, Liu Y, Fulton EA. A global estimate of carbon stored in the world's mountain grasslands and shrublands, and the implications for climate policy. Glob Environ Chang.; 2014;28: 14–24. doi:10.1016/J.GLOENVCHA.2014.05.008

145. Sarvašová Z, Cienciala E, Beranová J, Vančo M, Ficko A, Pardos M. Pôvodná Práca – original PaPer Analysis of governance systems applied in multifunctional forest management in selected European mountain regions. Lesn Cas For J. 2014;60: 159–167. doi:10.2478/forj-2014-0017

146. Hill BH, Kolka RK, McCormick FH, Starry MA. A synoptic survey of ecosystem services from headwater catchments in the United States. Ecosyst Serv.; 2014;7: 106–115. doi:10.1016/J.ECOSER.2013.12.004

147. Fontana V, Radtke A, Walde J, Tasser E, Wilhalm T, Zerbe S, et al. What plant traits tell us: Consequences of land-use change of a traditional agro-forest system on biodiversity and ecosystem service provision. Agric Ecosyst Environ.; 2014;186: 44–53. doi:10.1016/J.AGEE.2014.01.006

148. Homolová L, Schaepman ME, Lamarque P, Clevers JGPW, de Bello F, Thuiller W, et al. Comparison of remote sensing and plant trait-based modelling to predict ecosystem services in subalpine grasslands. Ecosphere.; 2014;5: art100. doi:10.1890/ES13-00393.1

149. Rocca ME, Brown PM, MacDonald LH, Carrico CM. Climate change impacts on fire regimes and key ecosystem services in Rocky Mountain forests. For Ecol Manage.; 2014;327: 290–305. doi:10.1016/J.FORECO.2014.04.005

150. Bereczki K, Ódor P, Csóka G, Mag Z, Báldi A. Effects of forest heterogeneity on the efficiency of caterpillar control service provided by birds in temperate oak forests. For Ecol Manage.; 2014;327: 96–105. doi:10.1016/J.FORECO.2014.05.001

151. Osuri AM, Kumar VS, Sankaran M. Altered stand structure and tree allometry reduce carbon storage in evergreen forest fragments in India's Western Ghats. For Ecol Manage.; 2014;329: 375–383. doi:10.1016/J.FORECO.2014.01.039

152. Roche LM, O'Geen AT, Latimer AM, Eastburn DJ. Montane meadow hydropedology, plant community, and herbivore dynamics. Ecosphere.; 2014;5: art150. doi:10.1890/ES14-00173.1

153. Ryffel AN, Rid W, Grêt-Regamey A. Land use trade-offs for flood protection: A choice experiment with visualizations. Ecosyst Serv.; 2014;10: 111–123. doi:10.1016/J.ECOSER.2014.09.008

154. Bingham A, Porter E. Ozone effects on two ecosystem services at Great Smokey Mountains National Park, USA. Parks. 2015;32: 71–79.

155. Grili G, Nikodinoska N, Paletto A, De Meo I. Stakeholders preferences and Economic Value of forest Ecosystem Services: An Example in the Italian Alps. Balt For. 2015;21: 298–307.

156. Tian N, Poudyal N, Hodges D, Young T, Hoyt K. Understanding the Factors Influencing Nonindustrial Private Forest Landowner Interest in Supplying Ecosystem Services in Cumberland Plateau, Tennessee. Forests. Multidisciplinary Digital Publishing Institute; 2015;6: 3985–4000. doi:10.3390/f6113985

157. Li X-W, Li M-D, Dong S-K, Shi J-B. Temporal-spatial changes in ecosystem services and implications for the conservation of alpine rangelands on the Qinghai-Tibetan Plateau. Rangel J.; 2015;37: 31. doi:10.1071/RJ14084

158. Li Y, Deng H, Dong R. Prioritizing protection measures through ecosystem services valuation for the Napahai Wetland, Shangri-La County, Yunnan Province, China. Int J Sustain Dev World Ecol.; 2015;22: 142–150. doi:10.1080/13504509.2014.926298

159. Sinha B, Mishra S. Ecosystem services valuation for enhancing conservation and livelihoods in a sacred landscape of the Indian Himalayas. Int J Biodivers Sci Ecosyst Serv Manag.; 2015;11: 156–167. doi:10.1080/21513732.2015.1030693

160. Ray R, S Chandran MD, Ramachandra T V. Hydrological importance of sacred forest fragments in Central Western Ghats of India. Trop Ecol. 2015;56: 87–99.

161. Rodríguez N, Armenteras D, Retana J. National ecosystems services priorities for planning carbon and water resource management in Colombia. Land use policy.; 2015;42: 609–618. doi:10.1016/J.LANDUSEPOL.2014.09.013

162. Bhatta LD, van Oort BEH, Stork NE, Baral H. Ecosystem services and livelihoods in a changing climate: Understanding local adaptations in the Upper Koshi, Nepal. Int J Biodivers Sci Ecosyst Serv Manag.; 2015;11: 145–155. doi:10.1080/21513732.2015.1027793

163. Deshpande K, Kelkar N. How Do Fruit Bat Seed Shadows Benefit Agroforestry? Insights from Local Perceptions in Kerala, India. Biotropica.; 2015;47: 654–659. doi:10.1111/btp.12275

164. Ye Y, Zhang J, Chen L, Ouyang Y, Parajuli P. Dynamics of ecosystem service values in response to landscape pattern changes from 1995 to 2005 in Guangzhou, southern China. Appl Ecol Environ Res. 2015;13: 21–36. doi:10.15666/aeer/1301_021036

165. Andreopoulos D, Damigos D, Comiti F, Fischer C. Estimating the non-market benefits of climate change adaptation of river ecosystem services: A choice experiment application in the Aoos basin, Greece. Environ Sci Policy.; 2015;45: 92–103. doi:10.1016/J.ENVSCI.2014.10.003

166. Paletto A, Geitner C, Grilli G, Hastik R, Pastorella F, Rodrìguez Garcìa L. Mapping the value of ecosystem services: A case study from the Austrian Alps. Ann For Res. 2015;58. doi:10.15287/afr.2015.335

167. Bastian O, Stein C, Lupp G, Behrens J, Renner C, Grunewald K. The appreciation of nature and landscape by tourism service providers and visitors in the Ore Mountains (Germany). Landsc Online. 2015; 1–23. doi:10.3097/LO.201541

168. Zhu J, Zhou Y, Wang S, Wang L, Wang F, Liu W, et al. Multicriteria decision analysis

for monitoring ecosystem service function of the Three-River Headwaters region of the Qinghai-Tibet Plateau, China. Environ Monit Assess.; 2015;187: 355. doi:10.1007/s10661-015-4523-5

169. Shimada D. Multi-level natural resources governance based on local community: A case study on semi-natural grassland in Tarōji, Nara, Japan. Int J Commons. 2015;9: 486. doi:10.18352/ijc.510

170. Cui Q, Wang X, Li C, Cai Y, Liu Q, Li R. Ecosystem service value analysis of CO2 management based on land use change of Zoige alpine peat wetland, Tibetan Plateau. Ecol Eng.; 2015;76: 158–165. doi:10.1016/J.ECOLENG.2014.03.035

171. Hashimoto S, Nakamura S, Saito O, Kohsaka R, Kamiyama C, Tomiyoshi M, et al. Mapping and characterizing ecosystem services of social–ecological production landscapes: case study of Noto, Japan. Sustain Sci.; 2015;10: 257–273. doi:10.1007/s11625-014-0285-1

172. Lopes LFG, dos Santos Bento JMR, Arede Correia Cristovão AF, Baptista FO. Exploring the effect of land use on ecosystem services: The distributive issues. Land use policy.; 2015;45: 141–149. doi:10.1016/J.LANDUSEPOL.2014.12.008

173. van Oort B, Bhatta LD, Baral H, Rai RK, Dhakal M, Rucevska I, et al. Assessing community values to support mapping of ecosystem services in the Koshi river basin, Nepal. Ecosyst Serv.; 2015;13: 70–80. doi:10.1016/J.ECOSER.2014.11.004

174. Cingolani AM, Poca M, Giorgis MA, Vaieretti MV, Gurvich DE, Whitworth-Hulse JI, et al. Water provisioning services in a seasonally dry subtropical mountain: Identifying priority landscapes for conservation. J Hydrol.; 2015;525: 178–187. doi:10.1016/J.JHYDROL.2015.03.041

175. Li C, Zheng H, Li S, Chen X, Li J, Zeng W, et al. Impacts of conservation and human development policy across stakeholders and scales. Proc Natl Acad Sci U S A. National Academy of Sciences; 2015;112: 7396–401. doi:10.1073/pnas.1406486112

176. Palacios-Agundez I, Onaindia M, Potschin M, Tratalos JA, Madariaga I, Haines-Young R. Relevance for decision making of spatially explicit, participatory scenarios for ecosystem services in an area of a high current demand. Environ Sci Policy.; 2015;54: 199–209. doi:10.1016/J.ENVSCI.2015.07.002

177. Häyhä T, Franzese PP, Paletto A, Fath BD. Assessing, valuing, and mapping ecosystem services in Alpine forests. Ecosyst Serv.; 2015;14: 12–23. doi:10.1016/J.ECOSER.2015.03.001

178. Crouzat E, Mouchet M, Turkelboom F, Byczek C, Meersmans J, Berger F, et al. Assessing bundles of ecosystem services from regional to landscape scale: insights from the French Alps. Diekötter T, editor. J Appl Ecol.; 2015;52: 1145–1155. doi:10.1111/1365-2664.12502

179. Bernués A, Rodríguez-Ortega T, Alfnes F, Clemetsen M, Eik LO. Quantifying the multifunctionality of fjord and mountain agriculture by means of sociocultural and economic valuation of ecosystem services. Land use policy.; 2015;48: 170–178. doi:10.1016/J.LANDUSEPOL.2015.05.022

180. Maroschek M, Rammer W, Lexer MJ. Using a novel assessment framework to evaluate protective functions and timber production in Austrian mountain forests under climate

change. Reg Environ Chang.; 2015;15: 1543–1555. doi:10.1007/s10113-014-0691-z

181. Pessacg N, Flaherty S, Brandizi L, Solman S, Pascual M. Getting water right: A case study in water yield modelling based on precipitation data. Sci Total Environ.; 2015;537: 225–234. doi:10.1016/J.SCITOTENV.2015.07.148

182. Crouzat E, Martín-López B, Turkelboom F, Lavorel S. Disentangling trade-offs and synergies around ecosystem services with the influence network framework: illustration from a consultative process over the French Alps. Ecol Soc.; 2016;21: art32. doi:10.5751/ES-08494-210232

183. Popa B. Forest ecosystem services valuation in different management scenarios. Balt For. 2016;138.

184. Lutz DA, Burakowski EA, Murphy MB, Borsuk ME, Niemiec RM, Howarth RB. Trade-offs between three forest ecosystem services across the state of New Hampshire, USA: timber, carbon, and albedo. Ecol Appl.; 2016;26: 146–161. doi:10.1890/14-2207

185. Martínez Pastur Pablo Peri María V Lencinas Marina García-Llorente Berta Martín-López GL, Martínez Pastur Á V Lencinas GM, Peri PL, García-Llorente M, Martín-López B. Spatial patterns of cultural ecosystem services provision in Southern Patagonia. Landsc Ecol. doi:10.1007/s10980-015-0254-9

186. Marrero HJ, Medan D, Zarlavsky GE, Torretta JP. Agricultural land management negatively affects pollination service in Pampean agro-ecosystems. Agric Ecosyst Environ.; 2016;218: 28–32. doi:10.1016/J.AGEE.2015.10.024

187. Zarandian A, Baral H, Yavari A, Jafari H, Stork N, Ling M, et al. Anthropogenic Decline of Ecosystem Services Threatens the Integrity of the Unique Hyrcanian (Caspian) Forests in Northern Iran. Forests. Multidisciplinary Digital Publishing Institute; 2016;7: 51. doi:10.3390/f7030051

188. Vergara-Tabares DL, Badini J, Peluc SI. Fruiting phenology as a “triggering attribute” of invasion process: Do invasive species take advantage of seed dispersal service provided by native birds? Biol Invasions.; 2016;18: 677–687. doi:10.1007/s10530-015-1039-4

189. Kindu M, Schneider T, Teketay D, Knoke T. Changes of ecosystem service values in response to land use/land cover dynamics in Munessa–Shashemene landscape of the Ethiopian highlands. Sci Total Environ.; 2016;547: 137–147. doi:10.1016/J.SCITOTENV.2015.12.127

190. Schneibel A, Stellmes M, Röder A, Finckh M, Revermann R, Frantz D, et al. Evaluating the trade-off between food and timber resulting from the conversion of Miombo forests to agricultural land in Angola using multi-temporal Landsat data. Sci Total Environ.; 2016;548–549: 390–401. doi:10.1016/J.SCITOTENV.2015.12.137

191. De Beenhouwer M, Geeraert L, Mertens J, Van Geel M, Aerts R, Vanderhaegen K, et al. Biodiversity and carbon storage co-benefits of coffee agroforestry across a gradient of increasing management intensity in the SW Ethiopian highlands. Agric Ecosyst Environ.; 2016;222: 193–199. doi:10.1016/J.AGEE.2016.02.017

192. Hastik R, Walzer C, Haida C, Garegnani G, Pezzutto S, Abegg B, et al. Using the “Footprint” Approach to Examine the Potentials and Impacts of Renewable Energy Sources in the European Alps. Mt Res Dev.; 2016;36: 130–140. doi:10.1659/MRD-

JOURNAL-D-15-00071.1

193. Cuni-Sanchez A, Pfeifer M, Marchant R, Burgess ND. Ethnic and locational differences in ecosystem service values: Insights from the communities in forest islands in the desert. Ecosyst Serv.; 2016;19: 42–50. doi:10.1016/J.ECOSER.2016.04.004

194. Shedayi AA, Xu M, Hussain F, Sadia S, Naseer I, Bano S. Threatened plant resources: Distribution and ecosystem services in the world's high elevation park of the karakoram ranges. Pakistan J Bot. 2016;48: 999–1012.

195. Gandarillas R. V, Jiang Y, Irvine K. Assessing the services of high mountain wetlands in tropical Andes: A case study of Caripe wetlands at Bolivian Altiplano. Ecosyst Serv.; 2016;19: 51–64. doi:10.1016/J.ECOSER.2016.04.006

196. Quintas-Soriano C, Castro AJ, Castro H, García-Llorente M. Impacts of land use change on ecosystem services and implications for human well-being in Spanish drylands. Land use policy.; 2016;54: 534–548. doi:10.1016/J.LANDUSEPOL.2016.03.011

197. Yu D, Han S. Ecosystem service status and changes of degraded natural reserves – A study from the Changbai Mountain Natural Reserve, China. Ecosyst Serv.; 2016;20: 56–65. doi:10.1016/J.ECOSER.2016.06.009

198. Carvalho-Santos C, Sousa-Silva R, Gonçalves J, Honrado JP. Ecosystem services and biodiversity conservation under forestation scenarios: options to improve management in the Vez watershed, NW Portugal. Reg Environ Chang.; 2016;16: 1557–1570. doi:10.1007/s10113-015-0892-0

199. Vásquez-Lavín F, Ibarnegaray V, Ponce Oliva R, Hernández Hernández J. Payment for Ecosystem Services in the Bolivian Sub-Andean Humid Forest. J Environ Dev.; 2016;25: 306–331. doi:10.1177/1070496516655838

200. Bagstad KJ, Reed JM, Semmens DJ, Sherrouse BC, Troy A. Linking biophysical models and public preferences for ecosystem service assessments: a case study for the Southern Rocky Mountains. Reg Environ Chang.; 2016;16: 2005–2018. doi:10.1007/s10113-015-0756-7

201. Zoderer BM, Lupo Stanghellini PS, Tasser E, Walde J, Wieser H, Tappeiner U. Exploring socio-cultural values of ecosystem service categories in the Central Alps: the influence of socio-demographic factors and landscape type. Reg Environ Chang.; 2016;16: 2033–2044. doi:10.1007/s10113-015-0922-y

202. Sarkki S, Ficko A, Grunewald K, Nijnik M. Benefits from and threats to European treeline ecosystem services: an exploratory study of stakeholders and governance. Reg Environ Chang.; 2016;16: 2019–2032. doi:10.1007/s10113-015-0812-3

203. Kline JD, Harmon ME, Spies TA, Morzillo AT, Pabst RJ, McComb BC, et al. Evaluating carbon storage, timber harvest, and habitat possibilities for a Western Cascades (USA) forest landscape. Ecol Appl. 2016;26: 2044–2059. doi:10.1002/eap.1358

204. Ezebilo EE. Economic value of a non-market ecosystem service: an application of the travel cost method to nature recreation in Sweden. Int J Biodivers Sci Ecosyst Serv Manag.; 2016; 1–14. doi:10.1080/21513732.2016.1202322

205. Egarter Vigl L, Schirpke U, Tasser E, Tappeiner U. Linking long-term landscape dynamics to the multiple interactions among ecosystem services in the European Alps. Landsc Ecol.; 2016;31: 1903–1918. doi:10.1007/s10980-016-0389-3

206. Zoderer BM, Tasser E, Erb K-H, Lupo Stanghellini PS, Tappeiner U. Identifying and mapping the tourists‘ perception of cultural ecosystem services: A case study from an Alpine region. Land use policy.; 2016;56: 251–261. doi:10.1016/J.LANDUSEPOL.2016.05.004

207. Austrheim G, Speed JDM, Evju M, Hester A, Holand Ø, Loe LE, et al. Synergies and trade-offs between ecosystem services in an alpine ecosystem grazed by sheep – An experimental approach. Basic Appl Ecol.; 2016;17: 596–608. doi:10.1016/J.BAAE.2016.06.003

208. Ferrari M, Geneletti D, Cayuela L, Orsi F, Benayas JMR. Analysis of Bundles and Drivers of Change of Multiple Ecosystem Services in an Alpine Region. J Environ Assess Policy Manag.; 2016;18: 1650026. doi:10.1142/S1464333216500265

209. Bremer LL, Farley KA, Oliver CD, Harden CP. Changes in carbon storage with land management promoted by payment for ecosystem services. Environ Conserv. 2016;43: 397–406. doi:10.1017/S0376892916000199

210. Jin X, Ma J, Cai T, Sun X. Non-use value assessment for wetland ecosystem service of Hongxing National Nature Reserve in northeast China. J For Res.; 2016;27: 1435–1442. doi:10.1007/s11676-016-0264-8

211. Peh KS-H, Thapa I, Basnyat M, Balmford A, Bhattarai GP, Bradbury RB, et al. Synergies between biodiversity conservation and ecosystem service provision: Lessons on integrated ecosystem service valuation from a Himalayan protected area, Nepal. Ecosyst Serv.; 2016;22: 359–369. doi:10.1016/J.ECOSER.2016.05.003

212. Brunner SH, Grêt-Regamey A. Policy strategies to foster the resilience of mountain social-ecological systems under uncertain global change. Environ Sci Policy.; 2016;66: 129–139. doi:10.1016/J.ENVSCI.2016.09.003

213. Dupire S, Bourrier F, Monnet J-M, Bigot S, Borgniet L, Berger F, et al. The protective effect of forests against rockfalls across the French Alps: Influence of forest diversity. For Ecol Manage.; 2016;382: 269–279. doi:10.1016/J.FORECO.2016.10.020
