# Supplementary material for: Nature’s contributions to people in mountains: A review
Source: PLoS One. 2019 Jun 11;14(6):e0217847. doi: 10.1371/journal.pone.0217847 (PMC6559649; doi:10.1371/journal.pone.0217847)
Supplement: S3 Text — (PDF) [file pone.0217847.s010.pdf]

S3 Text. Trends of research on nature's contributions to people in mountains in the time period of 2017-2018 based on a random sample of 10% of the screened papers (*n* = 310)

The search was applied to Abstract, Title and Keywords of published papers between 2017 and 2018. The search returned 413 papers. Based on the screening of titles and abstracts, we considered 310 papers for their assessment based on their full text. We randomly selected 10% of these 310 papers, i.e. 31 papers, for in-depth analyses in order to examine current patterns of ecosystem service research in mountains and to compare them with the ones found in the main study.

The research on ecosystem services in mountains in 2017-2018 follows a similar geographical pattern to that found in former years. China is the country with the highest number of publications (22.6%; *n* = 7) and Asia was the continent with the highest number of papers (32.1%; *n* = 10). European mountains were studied in 29.0% of publications (*n* = 9), followed by mountains in America (22.6%; *n* = 7) and by Africa (16.1%; *n* = 5), with Ethiopia being the only African country represented in the random sample.

Regarding methods, the patterns are similar to the research conducted in the main study (i.e. 1997-2016): biophysical assessment methods were the most applied in 2017-2018 (54.8% of publications; *n* = 17), followed by plural and integrated assessments (22.6%; *n* = 7), economic valuation (12.9%; *n* = 4) and socio-cultural approaches (9.7%; *n* = 3).

The research on ecosystem services in mountains has a similar pattern in 2017-2018 to that found in previous years (i.e. 1997-2016) with regards to the components of the IPBES framework addressed. In 2017-2018, the assessed publications on ecosystem services in mountains also covered the component of nature in 38.7% of publications (*n* = 12). Amongst

the organizational levels of biodiversity, 12.9% of publications (*n* = 4) evaluated the community level, while the remaining evenly focused on a functional diversity perspective (*n* = 3) and biodiversity in its broad sense (*n* = 3). Regarding the category of nature's contributions to people, 51.6% of publications (*n* = 16) assessed more than one category, while 38.7% (*n* = 12) assessed only regulating contributions, 6.4% (*n* = 2) assessed only non-material contributions and 3.2% (*n* = 1) focused only on material contributions. The regulating contributions that received the highest scientific attention were soil formation (*n* = 17), regulation of freshwater quantity (*n* = 16), climate change regulation (*n* = 14) and habitat maintenance (*n* = 13). Physical and psychological experiences (*n* = 13) and provision of food and feed (*n* = 12) were the non-material and material contributions receiving the highest attention, respectively.

Quality of life was only assessed in 19.4% of publications (*n* = 6) on ecosystem services in mountains, with the dimension of basic materials being the category receiving the highest scientific attention (*n* = 6).

Impacts of drivers of change on nature's contributions to people were assessed in 74.2% of publications (*n* = 23): 43.5% of publications (*n* = 10) assessed both direct and indirect drivers of change, 13.0% of publications (*n* = 3) assessed only direct drivers and 26.1% of publications (*n* = 6) assessed only indirect drivers. Therefore, the trends regarding research on drivers of change are consistent with the main study, with a decreasing number of publications assessing direct drivers of change and an increasing number of publications assessing the effect of indirect drivers of change. The direct drivers of change that continued to receive more scientific attention in 2017-2018 were land use change (87.5% of publications that analyzed direct drivers; *n* = 14) and climate change (50.0%; *n* = 8). Likewise, the impact of current conservation policies on nature's contributions to people continued to be the most studied indirect driver of change (76.5% of publications that analyzed indirect drivers; *n* = 13),

followed by the impact of markets (29.4% of publications on indirect drivers; n = 5), agricultural policies (29.4%; n = 5) and cultural change (29.4%; n = 5).

Finally, institutional responses to preserve biodiversity and nature's contributions to people in mountains were only addressed by 25.8% of publications (n = 8), with protected areas being the institutional response that received more attention (n = 4).

List of papers in-depth assessed in 2017-2018

1. Singh RK, Hussain SM, Riba T, Singh A, Padung E, Rallen O, et al. Classification and management of community forests in Indian Eastern Himalayas: implications on ecosystem services, conservation and livelihoods. Ecol Process. Springer Berlin Heidelberg; 2018;7: 27. doi:10.1186/s13717-018-0137-5

2. Shukla AK, Pathak S, Pal L, Ojha CSP, Mijic A, Garg RD. Spatio-temporal assessment of annual water balance models for upper Ganga Basin. Hydrol Earth Syst Sci. 2018;22: 5357–5371. doi:10.5194/hess-22-5357-2018

3. Tolessa T, Gessese H, Tolera M, Kidane M. Changes in Ecosystem Service Values in Response to Changes in Landscape Composition in the Central Highlands of Ethiopia. Environ Process. Springer International Publishing; 2018;5: 483–501. doi:10.1007/s40710-018-0326-3

4. Kim I, Arnhold S. Mapping environmental land use conflict potentials and ecosystem services in agricultural watersheds. Sci Total Environ. Elsevier; 2018;630: 827–838. doi:10.1016/J.SCITOTENV.2018.02.176

5. Schirpke U, Meisch C, Tappeiner U. Symbolic species as a cultural ecosystem service in the European Alps: insights and open issues. Landsc Ecol. Springer Netherlands;

2018;33: 711–730. doi:10.1007/s10980-018-0628-x

6. Fagan KE, Willcox E V., Willcox AS. Public attitudes toward the presence and management of bats roosting in buildings in Great Smoky Mountains National Park, Southeastern United States. Biol Conserv. Elsevier; 2018;220: 132–139. doi:10.1016/J.BIOCON.2018.02.004

7. Liu Z, Wang Y, Yu P, Tian A, Wang Y, Xiong W, et al. Spatial Pattern and Temporal Stability of Root-Zone Soil Moisture during Growing Season on a Larch Plantation Hillslope in Northwest China. Forests. Multidisciplinary Digital Publishing Institute; 2018;9: 68. doi:10.3390/f9020068

8. Delelegn YT, Purahong W, Blazevic A, Yitaferu B, Wubet T, Göransson H, et al. Changes in land use alter soil quality and aggregate stability in the highlands of northern Ethiopia. Sci Rep. Nature Publishing Group; 2017;7: 13602. doi:10.1038/s41598-017-14128-y

9. Resende FM, Fernandes GW, Andrade DC, Néder HD, Resende FM, Fernandes GW, et al. Economic valuation of the ecosystem services provided by a protected area in the Brazilian Cerrado: application of the contingent valuation method. Brazilian J Biol. Instituto Internacional de Ecologia; 2017;77: 762–773. doi:10.1590/1519-6984.21215

10. Schirpke U, Kohler M, Leitinger G, Fontana V, Tasser E, Tappeiner U. Future impacts of changing land-use and climate on ecosystem services of mountain grassland and their resilience. Ecosyst Serv. Elsevier; 2017;26: 79–94. doi:10.1016/J.ECOSER.2017.06.008

11. Muñoz MC, Schaefer HM, Böhning-Gaese K, Schleuning M. Importance of animal and plant traits for fruit removal and seedling recruitment in a tropical forest. Oikos. John Wiley & Sons, Ltd (10.1111); 2017;126: 823–832. doi:10.1111/oik.03547

12. Tolessa T, Senbeta F, Abebe T. Land use/land cover analysis and ecosystem services valuation in the central highlands of Ethiopia. For Trees Livelihoods. Taylor & Francis; 2017;26: 111–123. doi:10.1080/14728028.2016.1221780

13. Tolessa T, Senbeta F, Kidane M. The impact of land use/land cover change on ecosystem services in the central highlands of Ethiopia. Ecosyst Serv. Elsevier; 2017;23: 47–54. doi:10.1016/J.ECOSER.2016.11.010

14. Han H, Dong Y. Assessing and mapping of multiple ecosystem services in Guizhou Province, China. Trop Ecol. 2017;58: 331–346.

15. Fleischer P, Pichler V, Fleischer Jr P, Holko L, Máliš F, Gömöryová E, et al. Forest ecosystem services affected by natural disturbances, climate and land-use changes in the Tatra Mountains. Clim Res. 2017;73: 57–71. doi:10.3354/cr01461

16. Mina M, Bugmann H, Klopcic M, Cailleret M. Accurate modeling of harvesting is key for projecting future forest dynamics: a case study in the Slovenian mountains. Reg Environ Chang. Springer Berlin Heidelberg; 2017;17: 49–64. doi:10.1007/s10113-015-0902-2

17. Fu B, Xu P, Wang Y, Yan K, Chaudhary S. Assessment of the ecosystem services provided by ponds in hilly areas. Sci Total Environ. Elsevier; 2018;642: 979–987. doi:10.1016/J.SCITOTENV.2018.06.138

18. Voda M, Montes Y. DESCENDING MOUNTAIN ROUTES FUTURE: THE NORTH YUNGAS AND FĂGĂRAȘ GEOSYSTEM`S COMPARATIVE STUDY. Geogr Tech. 2018;11: 87–101. doi:10.21163/GT

19. Mekuria W, Wondie M, Amare T, Wubet A, Feyisa T, Yitaferu B. Restoration of degraded landscapes for ecosystem services in North-Western Ethiopia. Heliyon.

Elsevier; 2018;4: e00764. doi:10.1016/J.HELIYON.2018.E00764

20. Malek Ž, Zumpano V, Hussin H. Forest management and future changes to ecosystem services in the Romanian Carpathians. Environ Dev Sustain. Springer Netherlands; 2018;20: 1275–1291. doi:10.1007/s10668-017-9938-4

21. Pérez-Suárez M, Flores-Navarro MA, Martínez-Campos ÁR, Estrada-Flores JG, Chávez-Mejía MC. Impact of Extensive Grazing on Supporting and Regulating Ecosystem Services of Mountain Soils. Mt Res Dev. International Mountain Society; 2018;38: 125–134. doi:10.1659/MRD-JOURNAL-D-17-00103

22. Zhai J, Yang M, Li J, Cai M, Hou P, Cao W. Ecosystem assessment and protection effectiveness of a tropical rainforest region in Hainan Island, China. J Geogr Sci. 2018;28: 415–428. doi:10.1007/s11442-018-1481-1

23. Kokkoris IP, Drakou EG, Maes J, Dimopoulos P. Ecosystem services supply in protected mountains of Greece: setting the baseline for conservation management. Int J Biodivers Sci Ecosyst Serv Manag. 2018;14: 45–59. doi:10.1080/21513732.2017.1415974

24. Arias-Arévalo P, Martín-López B, Gómez-Baggethun E. Exploring intrinsic, instrumental, and relational values for sustainable management of social-ecological systems. Ecol Soc. 2017;22. doi:10.5751/ES-09812-220443

25. Bastian O, Syrbe R-U, Slavik J, Moravec J, Louda J, Kochan B, et al. Ecosystem services of characteristic biotope types in the Ore Mountains (Germany/Czech Republic). Int J Biodivers Sci Ecosyst Serv Manag. 2017;13: 51–71. doi:10.1080/21513732.2016.1248865

26. Bagstad KJ, Semmens DJ, Ancona ZH, Sherrouse BC. Evaluating alternative methods

for biophysical and cultural ecosystem services hotspot mapping in natural resource planning. Landsc Ecol. Springer Netherlands; 2017;32: 77–97. doi:10.1007/s10980-016-0430-6

27. Schmidt AH, Li Y, Tang Y. Unintended Side Effects of Conservation: A Case Study of Changing Land Use in Jiuzhaigou, Sichuan, China. Mt Res Dev. 2017;37: 56–65. doi:10.1659/mrd-journal-d-15-00056.1

28. Xiong Q, Xiao Y, Ouyang Z, Pan K, Zhang L, He X, et al. Bright side? The impacts of Three Gorges Reservoir on local ecological service of soil conservation in southwestern China. Environ Earth Sci. Springer Berlin Heidelberg; 2017;76. doi:10.1007/s12665-017-6588-7

29. Luo L, Pan Y, Zhao Y, Yu C, Wu J. Land management influences trade-offs and the total supply of ecosystem services in alpine grassland in Tibet, China. J Environ Manage. Elsevier Ltd; 2017;193: 70–78. doi:10.1016/j.jenvman.2017.02.008

30. Schuler LJ, Bugmann H, Snell RS. From monocultures to mixed-species forests: is tree diversity key for providing ecosystem services at the landscape scale? Landsc Ecol. Springer Netherlands; 2017;32: 1499–1516. doi:10.1007/s10980-016-0422-6

31. Schermer M, Schirpke U, Tasser E, Leitinger G, Tappeiner U, Stotten R, et al. Participative Spatial Scenario Analysis for Alpine Ecosystems. Environ Manage. Springer US; 2017;60: 679–692. doi:10.1007/s00267-017-0903-7
