# Supplementary material for: Nature’s contributions to people in mountains: A review
Source: PLoS One. 2019 Jun 11;14(6):e0217847. doi: 10.1371/journal.pone.0217847 (PMC6559649; doi:10.1371/journal.pone.0217847)
Supplement: S4 Text — (PDF) [file pone.0217847.s011.pdf]

S4 Text. Factoring out the effect of the number of papers on network indicators

We observed differences in the densities and the numbers of nodes of the networks at the four time periods (**Table 1**). The increase in densities and the numbers of nodes could be explained by the increasing number of publications over time, but we needed to test this explanation.

Table 1. Network indicators for each time period

Time period	Number of papers	Network density	Number of nodes
1997-2007	15	0.171	36
2008-2010	33	0.282	34
2011-2013	72	0.296	40
2014-2016	94	0.800	41

To understand how the number of papers in each time period influenced network density and the number of nodes, we built 10,000 random datasets of papers. Each dataset had a number of papers varying from 1 to 100. In each dataset, each paper could include a random set of topics among the list of topics in our original dataset. The probability of a given topic being considered in a paper depended on the topic and was estimated from our original dataset. After building the networks, we calculated indicators of each random networks and compared them to the indicators in our four observed networks.

Results showed that the numbers of nodes (i.e. the number of topics considered in the set of papers) were similar in our observations and in the random networks (**Fig 1**). In other terms, the increase in the number of nodes did not reflect more diverse research but only the increasing number of publications, as a larger number of publications is likely to cover a

larger number of topics. On the contrary, results showed that observed network densities were lower than in the random networks during the first time periods and higher during the last one (**Fig 2**). This means that papers in the first three time periods covered fewer pairs of topics than would be expected with a random selection of topics. In other terms, the most recent papers were more likely to cover more diverse combinations of topics.

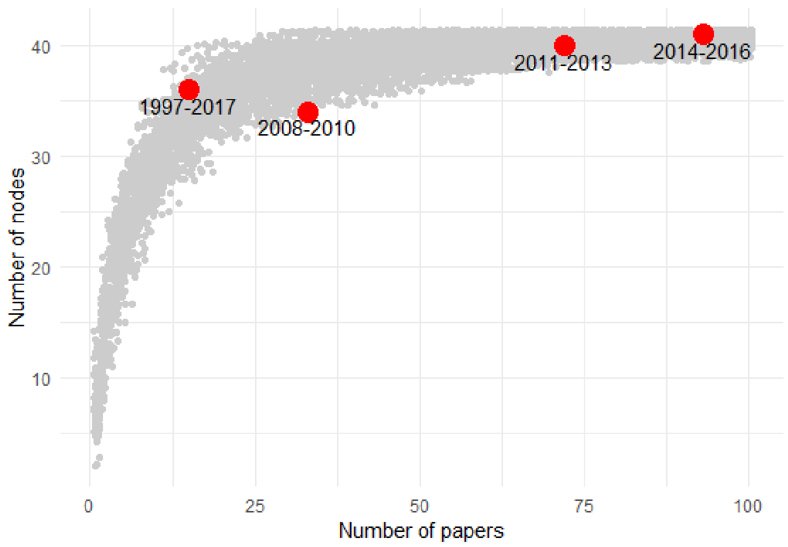

Figure 1. Effect of the number of papers on the number of nodes (i.e., topics) in random networks (grey cloud of dots) and in the four period of our dataset (red dots)

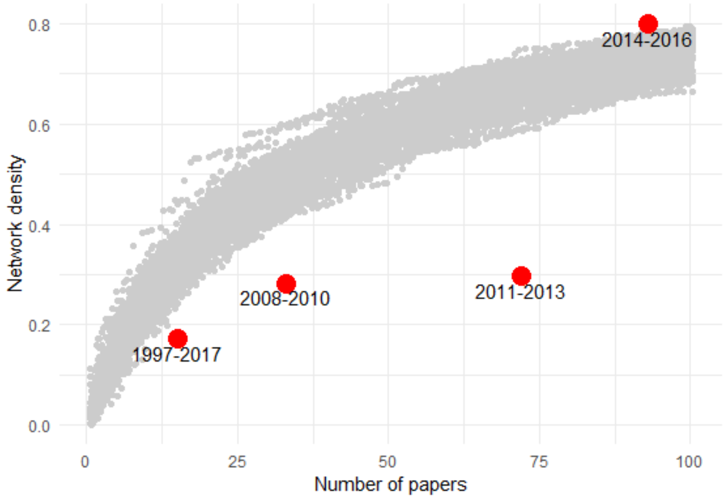

Figure 2. Effect of the number of papers on network density in random networks (grey cloud of dots) and in the four period of our dataset (red dots)
